# Synthesis and single-molecule magnet properties of a trimetallic dysprosium metallocene cation†

**DOI:** 10.1039/d1cc02139g

**Published:** 2021-05-28

**Authors:** Mian He, Fu-Sheng Guo, Jinkui Tang, Akseli Mansikkamäki, Richard A. Layfield

**Affiliations:** Department of Chemistry, School of Life Sciences, University of Sussex Brighton BN1 9QR UK R.Layfield@sussex.ac.uk; Changchun Institute of Applied Chemistry, Chinese Academy of Sciences, Renmin Street 5626 Changchun 130022 China; NMR Research Unit, University of Oulu, P.O. Box 8000 FI-90014 Finland Akseli.Mansikkamaki@oulu.fi

## Abstract

The dimetallic fulvalene-bridged dysprosium complex [{Dy(Cp*)(μ-BH_4_)}_2_(Fv^tttt^)] (1, Cp* = C_5_Me_5_) is converted into the trimetallic borohydride-bridged species [{Dy(Cp*)(Fv^tttt^)}_2_Dy(μ-BH_4_)_3_] (2). In turn, 2 is reacted with a silylium electrophile to give [{Dy(Cp*)(μ-BH_4_)(Fv^tttt^)}_2_Dy][B(C_6_F_5_)_4_] ([3][B(C_6_F_5_)_3_]), the first trimetallic dysprosocenium cation. Compound [**3**][B(C_6_F_5_)_3_] can also be formed directly from 1 by adding two equivalents of the electrophile. A three-fold enhancement in the effective energy barrier from 2 to 3 is observed and interpreted with the aid of *ab initio* calculations.

## Abstract 2

The first trimetallic dysprosocenium single-molecule magnet is described. This material signposts a polycationic dysprosium metallocene SMM with properties that could surpass those of leading systems.

## Graphical abstract

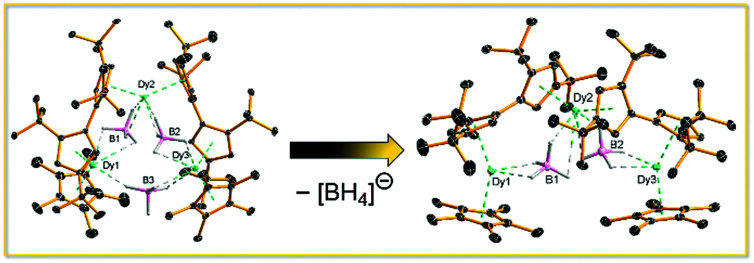

Achieving chemical control over magnetic relaxation times is a defining challenge in single-molecule magnetism. The multitude of physical processes that govern relaxation in single-molecule magnets (SMMs) arise from complex phenomena within individual molecules and their interactions with the lattice. To target slower relaxation at higher temperatures, several innovative strategies based on f- and d-block coordination and organometallic chemistry have been developed.^1–4^

The trivalent cation of dysprosium is the current *Drosophila* of single-molecule magnetism.^5,6^ In particular, dysprosocenium SMMs, *i.e.* [(η^5^-Cp^R^)_2_Dy]^+^, where Cp^R^ is a substituted cyclopentadienyl ligand, provide striking illustrations of what can be achieved.^7–11^ These cations have pseudo-axial geometries and contain no equatorial ligands; their properties include very large effective energy barriers to reversal of the magnetization (*U*_eff_) and magnetic hysteresis at unprecedentedly high temperatures (*T*_B_ = 60–80 K), with non-zero coercivity. Dysprosium metallocene cations also play an important signposting role that could allow SMM performance to be improved further. It has been shown that strict axial symmetry is not a prerequisite for observing large *U*_eff_ and *T*_B_ parameters, and that varying the Cp^R^ substituents can change the geometry in ways that impact on the SMM properties.^9^ Dysprosium metallocene cations have also served as case studies for demonstrating the importance of phonon modes and how the coupling of these modes to the spin facilitates activated magnetic relaxation processes.^7,11^

As our understanding of monometallic dysprosocenium SMMs improves, attention is turning to polymetallic analogues, in which exchange coupling adds to the complexity. Dimetallic dysprosocenium SMMs are known, typically with a weakly coordinating anion bridging the Dy^3+^ ions.^12–14^ Trimetallic dysprosocenium SMMs are unknown. Beyond the fundamental interest in triangular molecular magnets, additional motivation for targeting a system of this type is provided by the series of dysprosium Ising spin triangles, in which the remarkable phenomenon of toroidal magnetism was discovered.^15,16^ Having established a route to dimetallic dysprosocenium SMMs using the binucleating fulvalenyl (*i.e.* dicyclopentadienyl) ligand [1,1′,3,3′-(C_5_^*t*^Bu_2_H_2_)_2_]^2−^ (Fv^tttt^),^14^ we aimed to use this platform to synthesize a trimetallic analogue. The target compound was isolated using two routes. Firstly, dimetallic [{Dy(Cp*)(μ-BH_4_)}_2_(Fv^tttt^)] (**1**, Cp* = C_5_Me_5_) was reacted with two equivalents of the electrophile [(Et_3_Si)_2_(μ-H)][B(C_6_F_5_)_4_] to give [{Dy(Cp*)(μ-BH_4_)(Fv^tttt^)}_2_Dy][B(C_6_F_5_)_4_] ([**3**][B(C_6_F_5_)_3_]). Secondly, since it has been shown that nucleophilic reagents can remove borohydride ligands from early transition metal sandwich-type complexes,^17^ the reaction of **1** with PMe_3_ or ^*n*^BuLi was undertaken, allowing [{Dy(Cp*)(Fv^tttt^)}_2_Dy(μ-BH_4_)_3_] (**2**) to be isolated, which was then reacted with [(Et_3_Si)_2_(μ-H)][B(C_6_F_5_)_4_] to give **3** (Scheme 1).

**Scheme 1:**
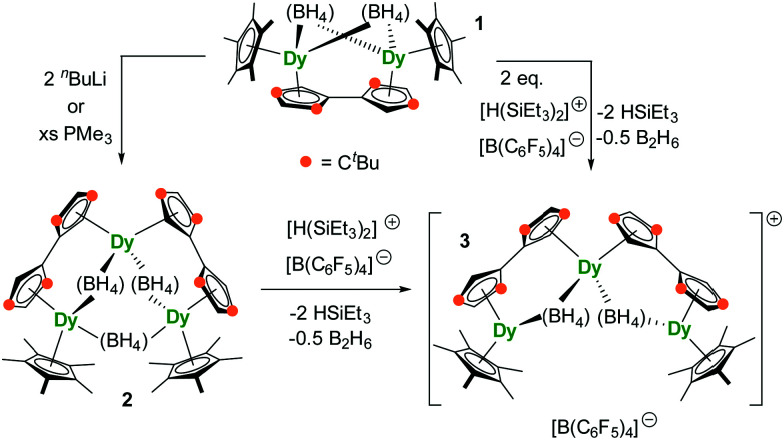
Synthesis of **2** and [**3**][B(C_6_F_5_)_4_].

Compounds **2** and [**3**][B(C_6_F_5_)_4_] were isolated in yields of 42% and 28%, respectively. The molecular structure of **2** consists of a Dy_3_ core with each pair of dysprosium atoms bridged by a borohydride ligand (Fig. 1 and Table S1, ESI†). In addition, Dy1 and Dy2 are bridged by an η^5^ : η^5^-fulvalenyl ligand, as are Dy2 and Dy3. Dy1 and Dy3 are also coordinated by an η^5^-Cp* ligand. Each dysprosium therefore occupies a {Cp_2_Dy(μ-BH_4_)_2_} environment, with the metallocene subunits twisted relative to each other (Table S2, ESI†). The six Dy–Cp_cent_ distances (‘cent’ denotes the Cp centroid) lie in the range 2.382(3)–2.407(2) Å and the Cp–Dy–Cp bending angles at Dy1, Dy2 and Dy3 are 135.708(12), 139.205(11) and 132.835(7)°, respectively. The intramolecular Dy⋯Dy distances are 4.741(4), 4.715(3) and 5.685(3) Å for Dy1/Dy2, Dy2/Dy3 and Dy3/Dy1, respectively.

**Fig. 1:**
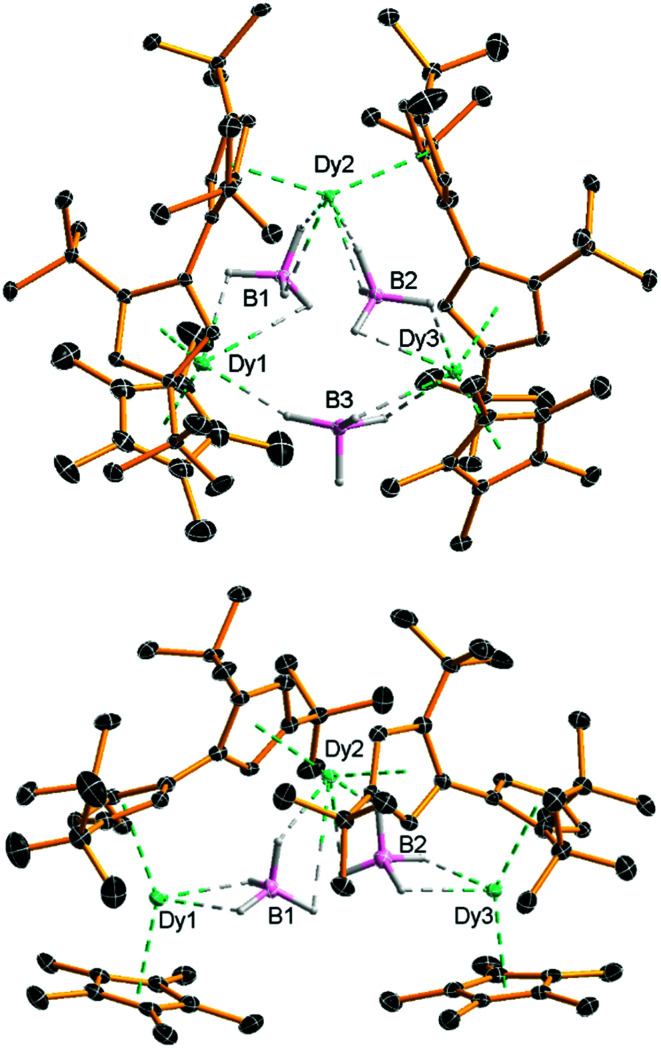
Molecular structures of **2** and the cation **3**. Thermal ellipsoids at 30% probability. For clarity, only the hydrogen atoms bound to boron are displayed.

The geometric constraints imposed on **2** by the fulvalenyl ligands bring Dy1 and Dy3 closer to Dy2, resulting in the relatively long Dy1⋯Dy3 separation. Consequently, the borohydride ligand bridging between Dy1 and Dy3 is exposed to attack by the electrophile [(Et_3_Si)_2_(μ-H)]^+^, resulting in formation of the V-shaped trimetallic cation **3** as the salt of [B(C_6_F_5_)_4_]^−^. Adding more than one equivalent of silylium electrophile to **2** or [**3**][B(C_6_F_5_)_4_] did not result in further reaction, presumably because the remaining borohydride ligands are protected by the bulk of the Fv^tttt^ and Cp* ligands. The Dy1⋯Dy2 and Dy2⋯Dy3 distances in **3** are, at 4.880(4) and 4.867(5) Å, significantly longer than in **2**, and the Dy1⋯Dy3 separation is now 7.908(1) Å (Fig. 1 and Table S3, ESI†). The Dy–Cp_cent_ distances range from 2.311(3) Å to 2.414(3) Å and are, on average, approximately 0.04 Å shorter than the analogous distances in **2**. Furthermore, the Cp–Dy–Cp angles in **3** are more obtuse than those in **2**, being 147.752(14), 147.578(14) and 149.159(16)° for Dy1, Dy2 and Dy3, respectively. The Dy⋯B distances in **2** and **3** are 2.741(4)–3.362(5) and 2.688(7)–2.980(7) Å, respectively, making them comparable to the analogous distances of 2.69 and 2.82 Å determined for the α- and β-forms of Dy(BH_4_)_3_.^18^ The FTIR spectra of **2** and **3** show absorptions in the region *ṽ* = 2185–2470 cm^−1^, corresponding to B–H stretches (Fig. S1 and S2, ESI†).

Based on the magneto-structural correlation developed for dysprosium metallocene SMMs,^19,20^ the effective energy barrier to reversal of the magnetization (*U*_eff_) for **3** should be larger than that for **2**. This is because the crystal fields experienced by the individual Dy^3+^ centres in the {(Cp^R^)_2_Dy} units in **3** are stronger and more axial, leading to larger crystal field splitting. Reducing the number of equatorial borohydride ligands should also be beneficial for the SMM properties of **3**. The real and imaginary components of the AC magnetic susceptibility, *χ*′ and *χ*′′, were measured in zero DC field as functions of temperature at various AC frequencies in the range *ν* = 1.0–1488 Hz, and as functions of frequency at various temperatures in the range 2–50 K for **2** (Fig. S6–S11, ESI†) and 2–67 K for [**3**][B(C_6_F_5_)_4_] (Fig. S13–S17, ESI†), respectively. Focusing on the *χ*′′(*ν*) data for **2**, maxima were observed from 10 K up to 46 K, with the position of the maxima shifting to higher frequencies with increasing temperature (Fig. 2). The *χ*′′(*ν*) data for [**3**][B(C_6_F_5_)_4_] are similar, but with maxima observed at 12–60 K. Evidently, the differences between the two chemically distinct dysprosium coordination sites in **2** and **3** (*i.e.* Dy1/Dy3 and Dy2) are not significant enough to allow separate relaxation processes to be resolved using a standard Magnetic Property Measurement System. Parabola-shaped Argand plots of *χ*′′(*χ*′) were obtained and fitted using Cole–Cole equations to obtain the relaxation time (*τ*) at each temperature for which a maximum was observed in the *χ*′′(*ν*) data (Fig. S10 and S17, ESI†). Good fits were obtained using α-parameters in the range 0.04 (*T* = 46 K) to 0.25 (*T* = 10 K) for **2**, and 0.05 (*T* = 60 K) to 0.26 (*T* = 12 K) for [**3**][B(C_6_F_5_)_4_] (Tables S4 and S5, ESI†). The variations in *α* indicate that *τ* extends over a wider range at lower temperatures.

**Fig. 2:**
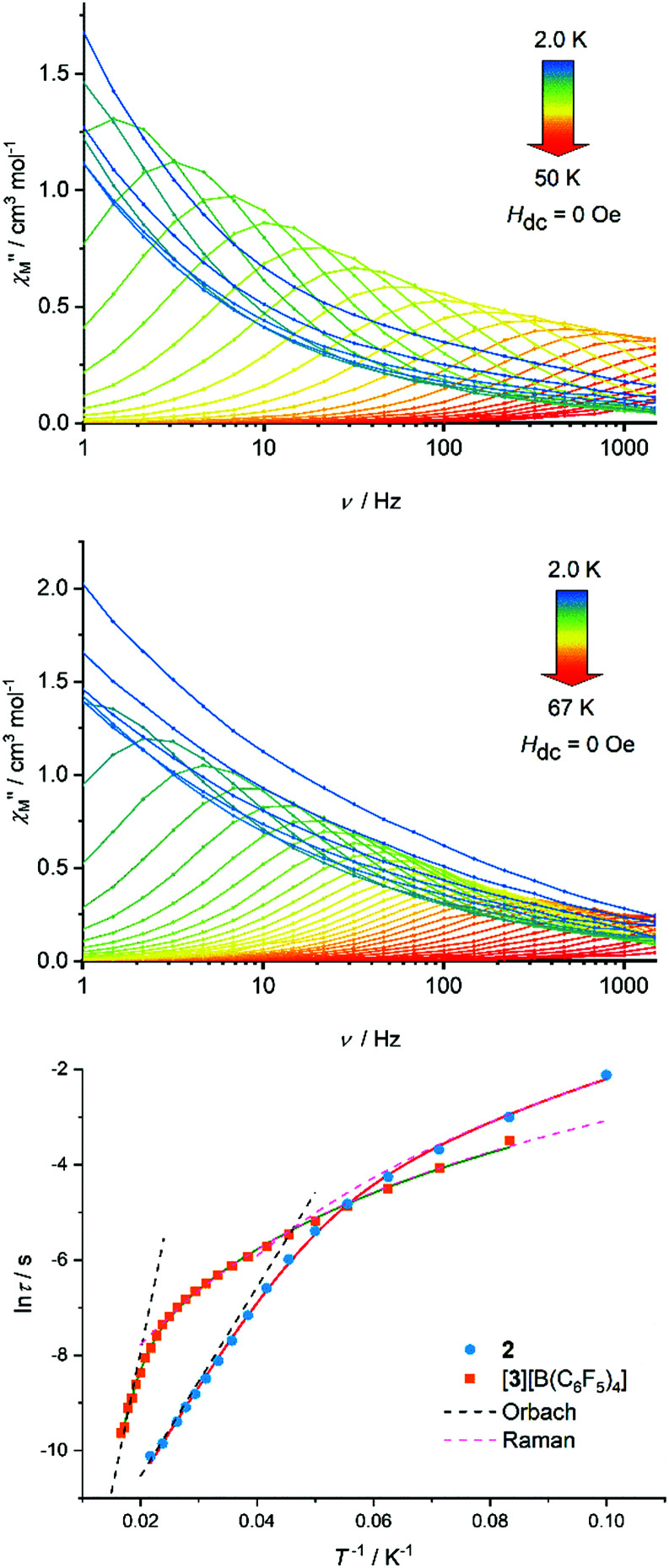
Imaginary component of the AC susceptibility as a function of frequency at the temperatures indicated for **2** (upper) and [**3**][B(C_6_F_5_)_4_] (middle). Temperature-dependence of the relaxation time for both compounds (lower). Solid lines represent fits to the data using the parameters stated in the text.

In the case of **2**, the dependence of ln *τ* on *T*^−1^ is roughly linear in the region 26–46 K, before deviations from linearity occur at lower temperatures (Fig. 2). A similar trend occurs for [**3**][B(C_6_F_5_)_4_] at 50–60 K before curvature is observed in the data. Qualitatively, these data suggest that thermally activated relaxation processes are dominant in these higher temperature regimes. The relaxation time does not become temperature independent for either compound in the range where maxima were observed in the *χ*′′(*ν*) data, suggesting that relaxation *via* quantum tunnelling of the magnetization (QTM) is inefficient and, hence, that relaxation *via* the Raman mechanism is important at lower temperatures.

Good fits of the relaxation time data were obtained using 
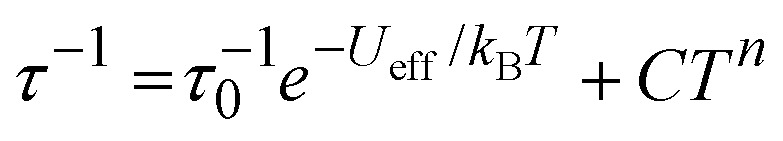
, where *τ*^−1^_0_ is the attempt time, *C* is the Raman coefficient and *n* is the Raman exponent. For **2**, the fit yielded *U*_eff_ = 138(4) cm^−1^, *τ*_0_ = 5.44(7) × 10^−7^ s, *C* = 8.03(4) × 10^−4^ s^−1^ K^−*n*^, *n* = 4.05(2). For [**3**][B(C_6_F_5_)_4_], the fit parameters are *U*_eff_ = 411(23) cm^−1^, *τ*_0_ = 4.16(2) × 10^−9^ s, *C* = 2.66(3) × 10^−4^ s^−1^ K^−*n*^, *n* = 2.92(1) (Fig. 2). Since **2** and **3** contain chemically distinct dysprosium environments, the parameters represent averages for each trimetallic species. However, the three-fold increase in *U*_eff_ from **2** to **3** is significant and fully consistent with the stronger, more-axial crystal field predicted for **3** based on the molecular structure.

Insight into the electronic structure of the individual Dy^3+^ centres in **2** and **3**, and the interactions between them, was obtained using multireference *ab initio* calculations. Both complexes consist of three dysprosium ions with strongly axial ground Kramers doublets (KDs), the axiality of which varies between the ions. In **2** and **3**, the ground KD of Dy2 shows the greatest axiality. The calculated local **g**-tensors are listed in Tables S6–S11 (ESI†). The transverse components of the **g**-tensors in the excited doublets for each Dy^3+^ ion in **2** and **3** gradually increase, as do the angles between the principal magnetic axes of the ground and excited KDs. In **2**, high axiality is retained up to the second excited KD in the case of Dy1 and Dy3, and up to the third excited KD in the case of Dy2. In **3**, significant deviations from axiality already occur in the first excited doublets of Dy1 and Dy3, whereas high axiality is retained up to the third excited KD in Dy2. The principal magnetic axes of the local ground KDs of each Dy^3+^ site in **2** and **3** are aligned roughly along the Cp–Dy–Cp axes and are shown in Fig. 3. In both complexes, the magnetic axis of the ground KD of the Dy2 ion lies roughly perpendicular relative to the other two axes. In **2**, the respective angles are 94.7° and 85.1°, and in the case of **3** they are 84.6° and 83.2°. This reduces the overall magnetization of the complexes in their ground state and precludes single-molecule toroic properties.

**Fig. 3:**
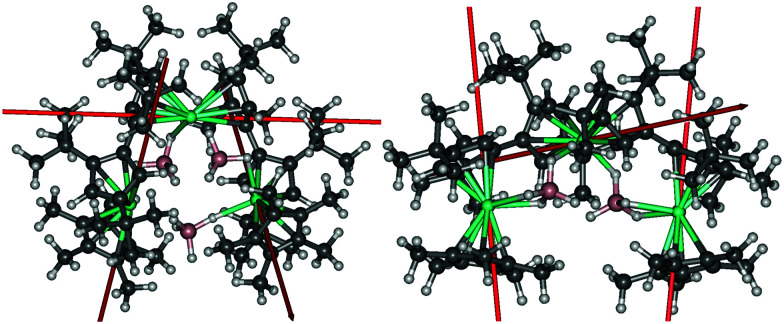
The principal magnetic axes of the local ground KDs of the Dy^3+^ ions in **2** (left) and **3** (right).

The bridging [BH_4_]^−^ anions do not allow efficient superexchange between the magnetic sites, and the interactions are dominated by dipolar coupling. An attempt was made to fit the exchange parameters using the Lines modes and the experimental magnetic susceptibility.^21,22^ However, the Lines exchange would make such a small contribution compared to the dipolar exchange that it was not possible to reliably fit the parameters. Thus, the exchange interaction was modelled considering only the dipolar coupling, which is in any case the dominant interaction (Fig. S20, ESI†). Projecting the dipolar coupling to an Ising-type pseudospin coupling Hamiltonian, acting on pseudospins of the ground KDs of each site, the nearest-neighbour exchange parameters are *J*_12_ = 1.81 cm^−1^ and *J*_23_ = −1.71 cm^−1^ for **2**, and *J*_12_ = −1.19 cm^−1^ and *J*_23_ = −1.25 cm^−1^ for **3**. The longer distance between Dy1 and Dy3 leads to a very weak interaction with *J*_13_ = 0.07 cm^−1^ for **2**, and for **3** the value cannot reliably be distinguished from numerical noise.

Due to the strong local axiality, weak exchange and dipolar interactions, the relaxation is driven by the local relaxation at each Dy^3+^ site. The point at which each local barrier is crossed can be estimated based on analysis of the **g**-tensors and the qualitative effective barriers constructed from transition magnetic dipole moment matrix elements (Fig. S21, S22 and Tables S18–S29, ESI†).^23^ In **2**, the local barrier should be crossed at the second, third and second excited KDs at the earliest, and at the fourth, fifth and third excited KDs for Dy1, Dy2 and Dy3 at the latest, respectively. This would correspond to an effective barrier of 330–561 cm^−1^. However, the experimental barrier is much closer to the energy range of the first excited KDs, which lie at 151–190 cm^−1^. In **3**, the local barriers are crossed at the first, second and first excited KDs at the earliest, and at the third, fourth and third excited KDs for Dy1, Dy2 and Dy3 at the latest, respectively. This gives an energy range of 324–643 cm^−1^, consistent with the experimental value. Based on comparison between the calculated energies and the fitted barriers, the barrier is most likely crossed at the first excited KD in ions Dy1 and Dy3 and at the second excited KD in the case of Dy2.

The local barriers of each Dy^3+^ ion in **2** and **3** retain axiality in the lower doublets. Although the doublets up to the fifth-excited KD in the case of Dy2 in **2** show significant axiality, non-negligible transverse components in the **g**-tensors are present in the first excited KDs. This reduced axiality allows more efficient spin-phonon transitions as well as more efficient QTM due to the presence of the neighbouring Dy^3+^ ions. Indeed, the results suggest that the barriers are crossed at the first excited KDs or, at most, at the second excited KD in the case of Dy2 in **3**. The reason for the reduced axiality probably lies in the significant equatorial contribution to the crystal field (CF) arising from the [BH_4_]^−^ ligands. The *ab initio* CF parameters were calculated using a well-established methodology^24^ (Tables S12–S17, ESI†). Analysis of the rank *k* = 2 parameters reveals significant deviations from ideal axiality. The axial parameters *B*_20_ range from −361 cm^−1^ to −381 cm^−1^ in **2** and form 483 cm^−1^ to 553 cm^−1^ in **3**. There are also significant off-diagonal contributions, especially with Dy1 and Dy3, leading to significant loss of axiality. The magnitudes of the off-diagonal parameters |*B*_2±2_| vary from 26–104 cm^−1^ in **2** and 24–208 cm^−1^ in **3**.

In conclusion, a trimetallic dysprosium metallocene SMM consisting of bridging fulvalene and borohydride ligands has been synthesized and converted into the first example of a trimetallic dysprosocenium cation. The local geometry changes that occur upon removal of a borohydride ligand from **2** to give the cation **3** equate to stronger, more axial crystal fields, ultimately resulting in a three-fold increase in the effective energy barrier. *Ab initio* calculations reveal different energies for the crystal field split states of the individual dysprosium ions in **2** and **3**, consistent with their slightly different composition. The non-negligible equatorial crystal fields provided by the [BH_4_]^−^ ligands limit the barrier height; hence, our on-going work will focus on ways of removing these ligands in order to isolate a purely axial dysprosocenium polycation.

We thank the University of Sussex, the ERC (CoG 646740), the EPSRC (EP/M022064/1), the Academy of Finland (project 332294) the National Natural Science Foundation of China (21525103, 21871247) and the University of Oulu (Kvantum Institute). Computational resources were provided by CSC-IT Center for Science in Finland and the Finnish Grid and Cloud Infrastructure (persistent identifier urn:nbn:fi:research-infras-2016072533). J. T. and R. A. L. thank the Royal Society for a Newton Advanced Fellowship (NA160075).

## Conflicts of interest

There are no conflicts to declare.

## Supplementary Material

CC-057-D1CC02139G-s001

CC-057-D1CC02139G-s002
