# Supplementary material for: Synthesis and single-molecule magnet properties of a trimetallic dysprosium metallocene cation
Source: Chem Commun (Camb). 2021 May 28;57(52):6396–9. doi: 10.1039/d1cc02139g (PMC8240697; doi:10.1039/d1cc02139g)
Supplement: CC-057-D1CC02139G-s001 [file CC-057-D1CC02139G-s001.pdf]

Electronic Supplementary Material (ESI) for ChemComm.
This journal is © The Royal Society of Chemistry 2021

Synthesis and Single-Molecule Magnet Properties of a Trimetallic Dysprosium Metallocene Cation

Mian He,[a] Fu-Sheng Guo,[a] Jinkui Tang,[b] Akseli Mansikkamäki,*[c] Richard A. Layfield*[a]

[a] Department of Chemistry, School of Life Sciences, University of Sussex, BN1 9QR, U.K.

[b] State Key Laboratory of Rare Earth Resource Utilization, Changchun Institute of Applied Chemistry, Chinese Academy of Sciences, Changchun 130022, P.R. China.

[c] NMR Research Unit, University of Oulu, P.O. Box 8000, FI-90014, Finland.

General synthetic procedures

All reactions were carried out under rigorous anaerobic and anhydrous conditions using argon or nitrogen atmospheres and standard Schlenk or glove-box techniques. Solvents were refluxed over an appropriate drying agent for a minimum of three days (molten potassium for toluene, THF, benzene-D_6, Na/K alloy for hexane) before being distilled, degassed and stored in ampoules over activated 4 Å molecular sieves. Glass-coated stirrer bars were used for each reaction. Elemental analyses were carried out at Mikroanalytisches Labor Pascher company (Germany). IR spectra were collected on a Bruker Alpha FTIR spectrometer fitted with a Platinum ATR module. A literature procedure was used to synthesize $[\{Dy(Cp^*)(\mu\text{-}BH_4)\}_2(Fv^{tttt})]$. [1]

Synthesis of $[\{Dy(Cp^*)(Fv^{tttt})\}_2Dy(\mu\text{-}BH_4)_3]$ (2)

A solution of $[\{Dy(Cp^*)(\mu\text{-}BH_4)\}_2(Fv^{tttt})]$ (300 mg, 0.30 mmol) in hexane (20 mL) was cooled to 0°C and nBuLi (2.5 M in hexane, 243 μL, 0.60 mmol)) was added dropwise. The resulting suspension was warmed to room temperature and stirred overnight, during which time the yellow solution became lighter in colour. The suspension was filtered, the residue was washed with hexane (2 × 10 ml) and the filtrate and washings were combined. A light-yellow solution was obtained and the solvent was removed slowly under vacuum until a crystalline precipitate formed. Storage at –40 °C for two days produced pale-yellow crystals of **2**. The crystals were washed with cold hexane, re-dissolved in warm hexane and stored at –40 °C, which produced crystals of suitable quality for analysis by single-crystal X-ray diffraction. Isolated yield = 105 mg, 42 %.

We also found that compound **2** can be obtained by using excess of PMe_3 (ca. 1 mL) instead of n-BuLi, Isolated yield = 80 mg, 32 %.

Elemental analysis found (calcd.) % for $C_{72}H_{122}B_3Dy_3\cdot C_6H_{14}$ (hexane): C 58.70 (58.78); H 8.55 (8.60).

IR spectrum ($\tilde{\nu}$/cm^{-1}): 2953s, 2928w, 2901m, 2860m, 2438m, 2263s, 2213m, 1481w, 1459s, 1392m, 1359s, 1304m, 1269w, 1229s, 1199w, 1152w, 1126w, 1111w, 1087s, 1058w, 1023m, 957w, 928w, 858s, 801m, 703m, 672m, 620w, 594w, 571w, 549m, 505w, 430m.

Synthesis of $[\{Dy(Cp^*)(\mu\text{-}BH_4)(Fv^{tttt})\}_2Dy][B(C_6F_5)_4]$ ([3][$B(C_6F_5)_3$])

Solid $[(Et_3Si)_2(\mu\text{-}H)][B(C_6F_5)_4]$ (28 mg, 0.031 mmol) was added to a solution of **2** (50 mg, 0.031 mmol) in hexane (10 mL) and the resulting suspension was stirred overnight. A yellow powder formed. The hexane was removed, the residue was washed with hexane (5 × 10 ml) and the resulting yellow powder dried under vacuum. Dissolving the powder in 1,2-dichlorobenzene (5 ml) and layering with hexane at room temperature produced, after several days, bright yellow crystals. The solvents were then decanted away and the crystals were washed

with hexane (3 × 5 ml). The crystallization process was repeated twice in order to obtain single crystals of [{Dy(Cp*)(μ-BH_4)(Fv^{tttt})}$_2$Dy][B(C_6F_5)$_4$] suitable for X-ray diffraction. Yield = 20 mg, 28 %.

We also found that [**3**][B(C_6F_5)$_3$] can be obtained by reacting [(Et_3Si)$_2$(μ-H)][B(C_6F_5)$_4$] in excess (184 mg, 0.20 mmol) with [{Dy(Cp*)(μ-BH_4)}$_2$(Fv^{tttt})] (99 mg, 0.10 mmol) in hexane. Yield = 46 mg, 42 %.

Elemental analysis found (calcd.) % for $C_{96}H_{118}B_3F_{20}Dy_3$: C 52.70 (53.09); H 5.28 (5.48).

IR spectrum ($\tilde{\nu}$/cm^{-1}): 2961m, 2905w, 2870w, 2470m, 2256m, 2185m, 1643m, 1512s, 1462s, 1393w, 1360m, 1317w, 1269m, 1234m, 1200w, 1083s, 1035m, 979s, 927w, 837s, 774s, 756s, 726w, 707w, 683s, 660s, 610m, 572m, 506w, 476w, 435m.

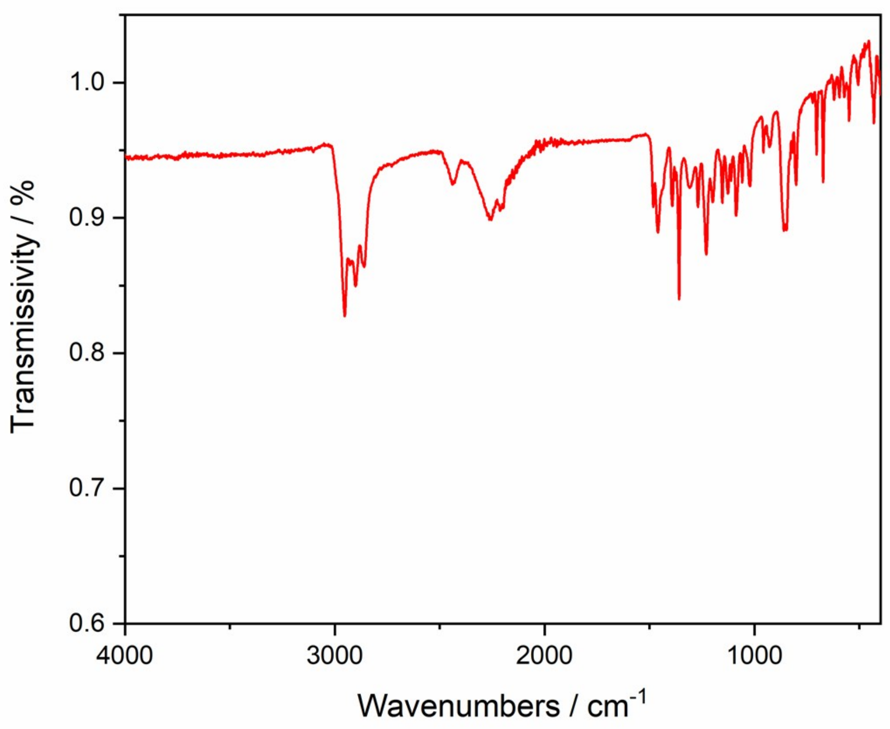

Fig. S1. Infrared spectrum of **2**.

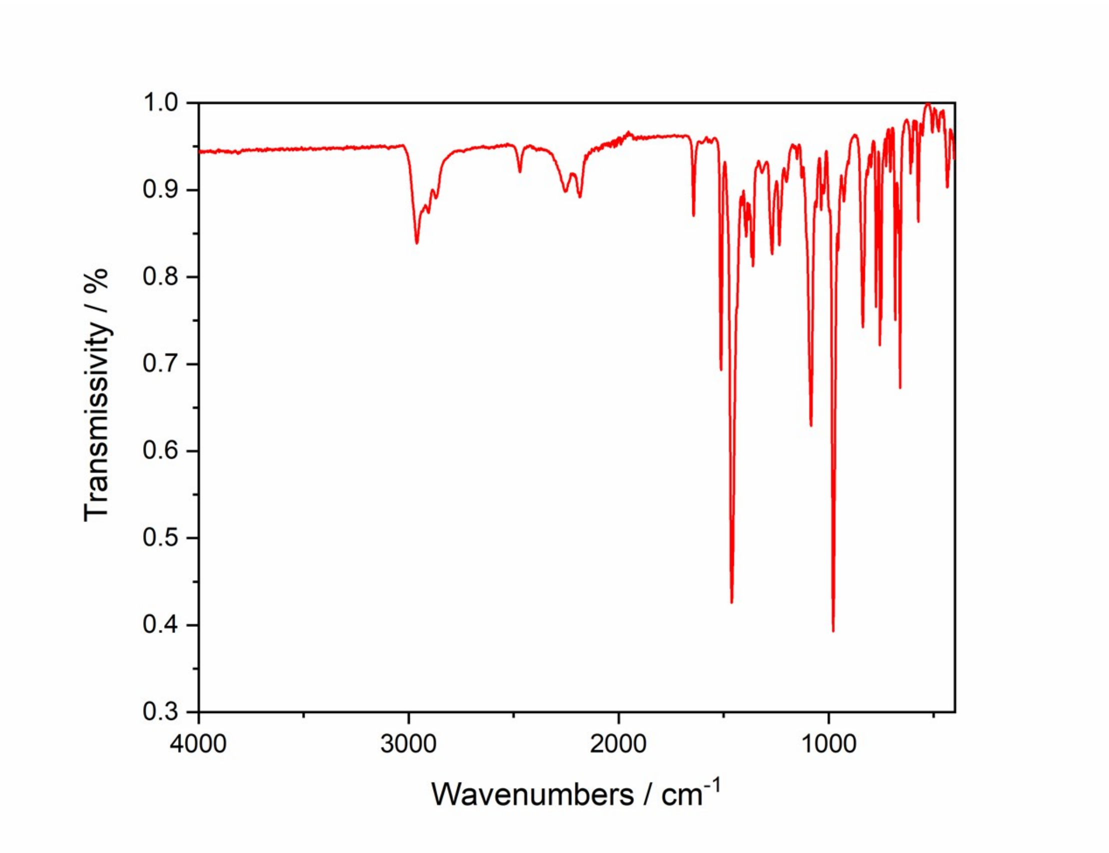

Fig. S2. Infrared spectrum of [**3**][B(C_6F_5)$_3$].

X-ray crystallography

Single-crystal X-ray diffraction measurements were carried out on an Agilent Gemini Ultra diffractometer with an Enhance Ultra (Cu *Kα*), equipped with an Eos CCD area detector, operating in *ω* scanning mode to fill the Ewald sphere. Control, integration and absorption corrections were processed with the CrysAlis[Pro] software. Crystals were mounted on MiTiGen loops from dried vacuum oil that had been kept over 4 Å molecular sieves in a glovebox under argon. Data were solved in Olex2 with SHELXT, using intrinsic phasing, and were refined with SHELXL using least squares minimisation. [2-4] The SQUEEZE program of PLATON was employed to deal with the disordered solvent molecules of compound **2**.

Table S1. Crystal data and structure refinement for **2** and [**3**][$B(C_6F_5)_3$].

**Compound reference**	**2**	[**3**][$B(C_6F_5)_3$]
CCDC ref. code	2077342	2077343
empirical formula	$C_{78}H_{136}B_3Dy_3$	$C_{102}H_{132}B_2Dy_3F_{20}$
formula weight	1593.79	2258.00
crystal system	monoclinic	triclinic
space group	$P2_1/n$	P–1
a/Å	14.14480(10)	14.2955(5)
b/Å	21.92880(10)	17.6222(7)
c/Å	26.0721(2)	19.9866(6)
α/°	90	80.945(3)
β/°	98.3090(10)	88.122(3)
γ/°	90	88.861(3)
Volume/Å^3	8002.11(9)	4969.0(3)
Z	4	2
Temperature/K	100	100
ρ_{calc} g/cm^3	1.323	1.509
F(000)	3268.0	2274.0
Reflections collected	55554	34352
Independent reflections	15365	17677
R_{int}	0.0814	0.0429
Goodness of fit on F^2	1.024	1.015
$R_1{}^a$	0.0407	0.0474
$R_w{}^b$	0.1060	0.1225

$^a R_1[I>2\sigma(I)] = \sum||F_o|-|F_c||/\sum|F_o|$; $^b R_w$[all data] $= [\sum\{w(F_o{}^2-F_c{}^2)^2\}/\sum\{w(F_o{}^2)^2\}]^{1/2}$

Table S2. Selected bond lengths (Å) and angles (°) for **2**.

Dy–C (fulvalene)	Dy1–C1: 2.709(4)	Dy2–C37: 2.663(4)
	Dy1–C2: 2.718(4)	Dy2–C38: 2.733(4)
	Dy1–C3: 2.654(4)	Dy2–C39: 2.682(4)
	Dy1–C4: 2.674(4)	Dy2–C40: 2.694(4)
	Dy1–C5: 2.638(4)	Dy2–C41: 2.620 (4)
	Dy2–C6: 2.668(4)	Dy3–C42: 2.725(4)
	Dy2–C7: 2.618(4)	Dy3–C43: 2.615(4)
	Dy2–C8: 2.689(4)	Dy3–C44: 2.663(4)
	Dy2–C9: 2.686(4)	Dy3–C45: 2.688(4)
	Dy2–C10: 2.747(4)	Dy3–C46: 2.775(4)
Dy–C (Cp*)	Dy1–C27: 2.688(5)	
	Dy1–C28: 2.669(4)	
	Dy1–C29: 2.650(5)	
	Dy1–C30: 2.641(5)	
	Dy1–C31: 2.666(5)	
	Dy3–C63: 2.686(4)	
	Dy3–C64: 2.665(4)	
	Dy3–C65: 2.663(4)	
	Dy3–C66: 2.683(4)	
	Dy3–C67: 2.679(4)	
Dy–centroid (fulvalene)	Dy1-centroid: 2.391(3)	Dy2-centroid2: 2.391(1)
	Dy2-centroid1: 2.394(1)	Dy3-centroid: 2.407(2)
Dy–centroid (Cp*)	Dy1-centroid: 2.382(3)	
	Dy3-centroid: 2.389(2)	
Dy···Dy	Dy1···Dy2: 4.741(4)	Dy2···Dy3: 4.715(3)
	Dy1···Dy3: 5.685(3)	
Dy···B	Dy1···B1: 2.741(4)	Dy2···B2: 2.895(4)
	Dy1···B3: 3.362(5)	Dy3···B2: 2.783(5)
	Dy2···B1: 2.943(4)	Dy3···B3: 2.887(5)
centroid-M-centroid	Dy1: 135.708(12)	Dy3: 132.835(7)
	Dy2: 139.205(11)	

Table S3. Selected bond lengths (Å) and angles (°) for [**3**][B(C_6F_5)$_3$].

Dy–C (fulvalene)	Dy1–C1: 2.672(5)<br>Dy1–C2: 2.719(5)<br>Dy1–C3: 2.629(5)<br>Dy1–C4: 2.587(5)<br>Dy1–C5: 2.562(5)<br>Dy2–C6: 2.723(5)<br>Dy2–C7: 2.626(5)<br>Dy2–C8: 2.686(5)<br>Dy2–C9: 2.659(5)<br>Dy2–C10: 2.726(5)	Dy2–C37: 2.730(5)<br>Dy2–C38: 2.757(5)<br>Dy2–C39: 2.680(5)<br>Dy2–C40: 2.691(5)<br>Dy2–C41: 2.645(5)<br>Dy3–C42: 2.639(5)<br>Dy3–C43: 2.598(5)<br>Dy3–C44: 2.630(5)<br>Dy3–C45: 2.613(5)<br>Dy3–C46: 2.668(5)
Dy–C (Cp*)	Dy1–C27: 2.606(5)<br>Dy1–C28: 2.620(5)<br>Dy1–C29: 2.605(5)<br>Dy1–C30: 2.590(5)<br>Dy1–C31: 2.606(5)<br>Dy3–C63: 2.603(5)<br>Dy3–C64: 2.601(5)<br>Dy3–C65: 2.606(5)<br>Dy3–C66: 2.609(6)<br>Dy3–C67: 2.630(5)	
Dy–centroid (fulvalene)	Dy1-centroid: 2.340(3)<br>Dy2-centroid1: 2.396(1)	Dy2-centroid2: 2.414(3)<br>Dy3-centroid: 2.336(3)
Dy–centroid (Cp*)	Dy1-centroid: 2.311(3)<br>Dy3-centroid: 2.314(3)	
Dy···Dy	Dy1···Dy2: 4.880(4)<br>Dy2···Dy3: 4.867(5)	Dy1···Dy3: 7.908(1)
Dy···B	Dy1···B1: 2.688(7)<br>Dy2···B1: 2.980(7)	Dy2···B2: 2.951(7)<br>Dy3···B2: 2.691(6)
centroid-M-centroid	Dy1: 147.752(14)<br>Dy2: 147.578(14)	Dy3: 149.159(16)

Magnetic property measurements

Magnetic susceptibility measurements were recorded on a Quantum Design MPMS-XL7 SQUID magnetometer equipped with a 7 T magnet. The samples were restrained in eicosane and sealed in 7 mm NMR tubes. Direct current (DC) magnetic susceptibility measurements were performed on crystalline samples in the temperature range 1.9-300 K using an applied field of 1000 Oe. The AC susceptibility measurements were performed in zero DC field. Diamagnetic corrections were made with Pascal's constants for all the constituent atoms. [5]

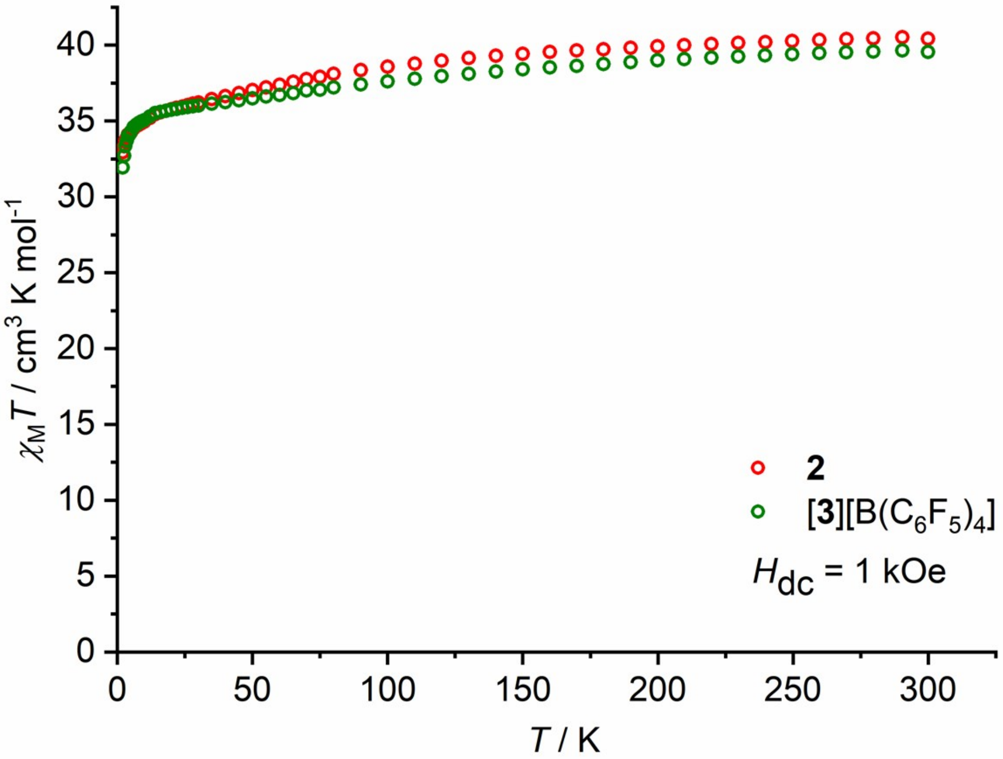

Fig. S3. Plot of $\chi_M T$ versus temperature for **2** and [**3**][B(C_6F_5)$_3$] in an applied field of 1 kOe. $\chi_M T$ (290 K) = 40.5 cm^3 K mol^{-1}, $\chi_M T$ (2.0 K) = 32.9 cm^3 K mol^{-1}.

The temperature-dependence of the molar magnetic susceptibility (χ_M) for **2** and [**3**][B(C_6F_5)$_4$] was measured in an applied DC field of 1000 Oe and in the temperature range 2-300 K. The values of $\chi_M T$ at 290 K were determined to be 40.5 cm^3 K mol^{-1} and 39.6 cm^3 K mol^{-1}, respectively, both of which are slightly lower than the theoretical value of 42.51 cm^3 K mol^{-1} for three weakly coupled Dy^{3+} ions. On decreasing the temperature, a gradual decrease in $\chi_M T$ was observed, followed by a more noticeable drop below 10 K. The behaviour of $\chi_M T(T)$ for both compounds is indicative of weak exchange coupling between the dysprosium ions, with thermal depopulation of the excited crystal field levels evident at the lowest measurement temperatures.

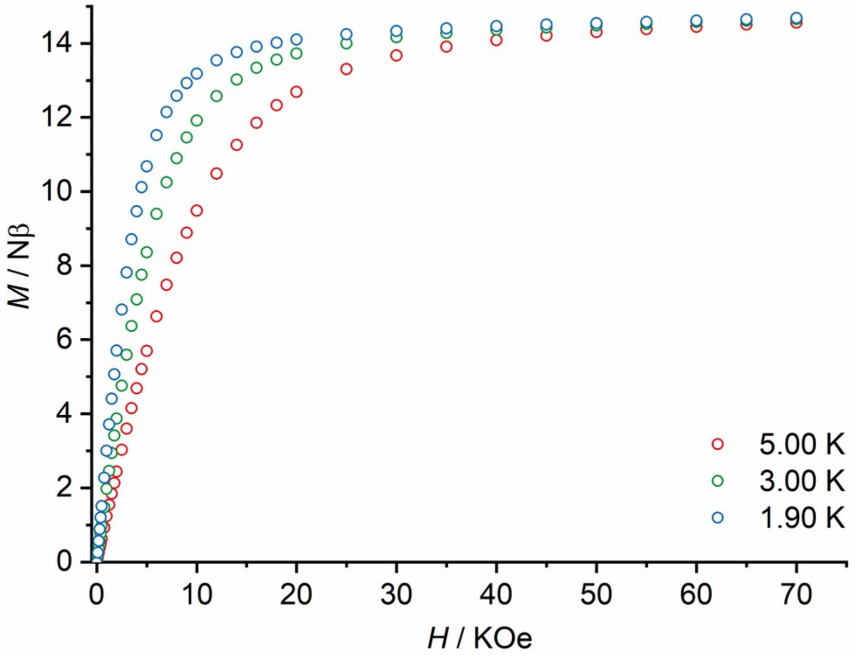

Fig. S4. Field-dependent isothermal magnetization for **2** at 1.9 K, 3.0 K and 5.0 K. The value of *M* at 1.9 K and 7 T is 14.6 Nβ.

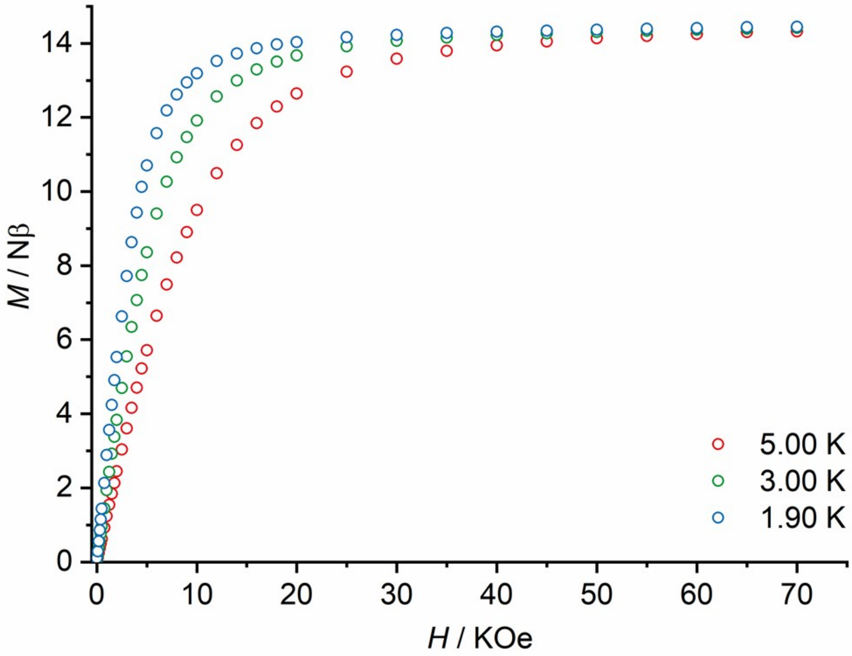

Fig. S5. Field-dependent isothermal magnetization for **2** at 1.9 K, 3.0 K and 5.0 K. The value of *M* at 1.9 K and 7 T is 14.3 Nβ.

At 1.9 K, the magnetization of **2** and [**3**][B(C_6F_5)$_4$] increases rapidly with increasing field up to approximately 3 T, before becoming essentially field independent reaching values of *M* = 14.6 mB and 14.3 mB at 7 T. The magnetization values are substantially lower than the theoretical maximum values of *M* = 30 mB for a trimetallic dysprosium complex, consistent with the presence of strong magnetic anisotropy.

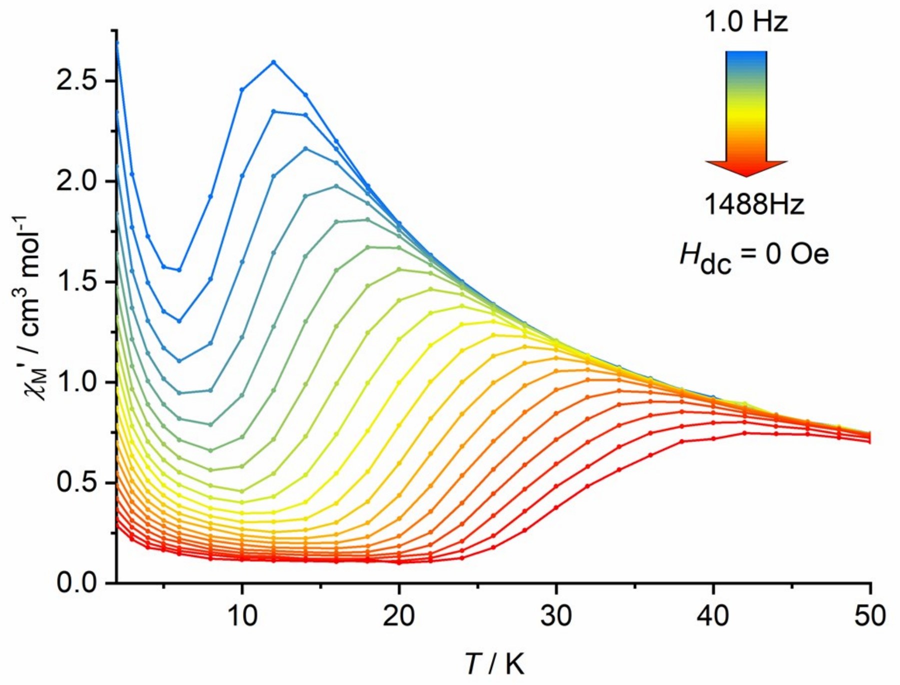

Fig. S6. Temperature dependence of the in-phase (χ'_M) AC susceptibility for **2** at various frequencies in the range 1.0 Hz (blue) to 1488 Hz (red) under zero DC field. Solid lines are a guide to the eye.

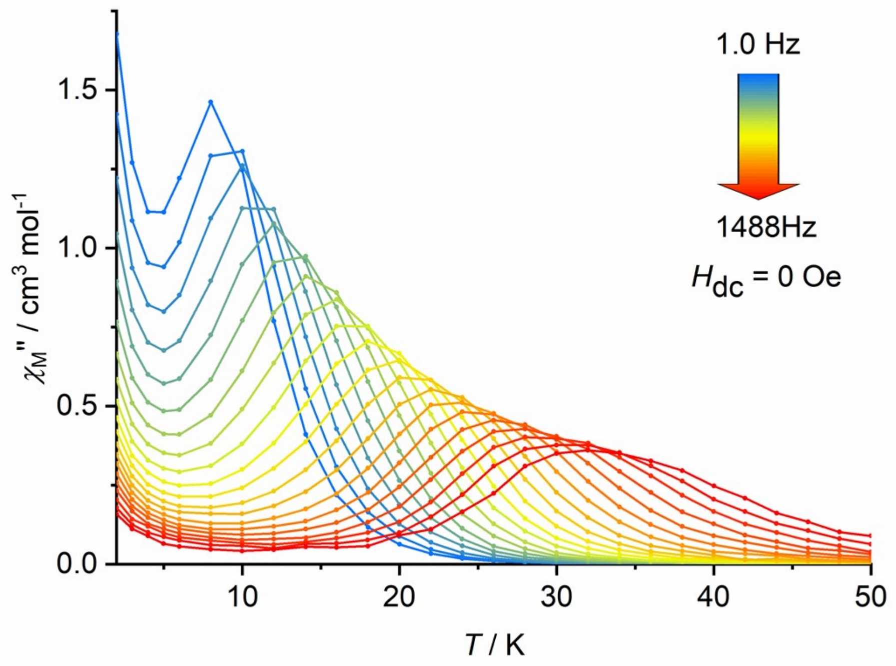

Fig. S7. Temperature dependence of the out-of-phase (χ''_M) AC susceptibility for **2** at various frequencies in the range 1.0 Hz (blue) to 1488 Hz (red) under zero DC field. Solid lines are a guide to the eye.

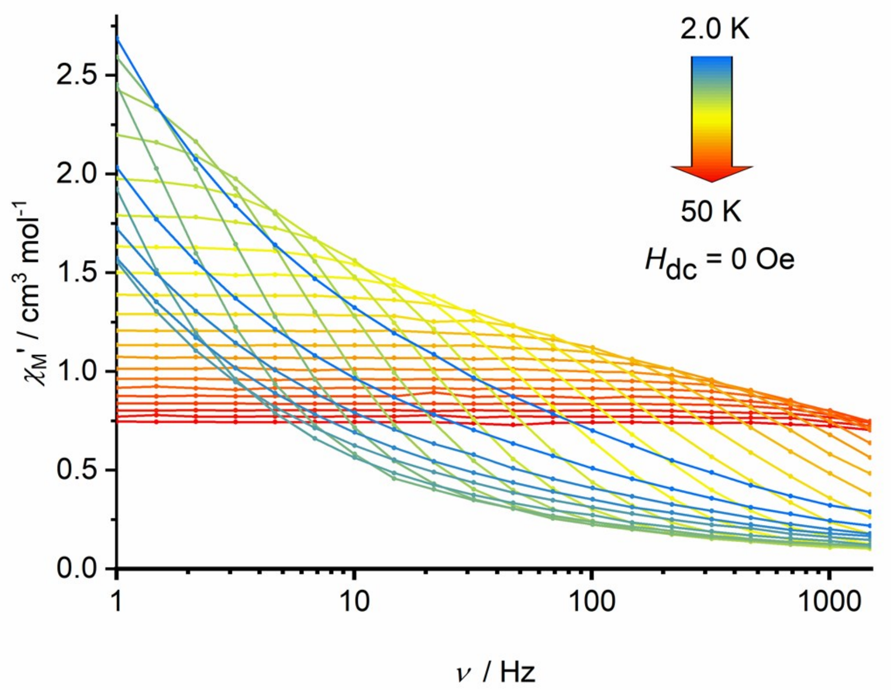

Fig. S8. Frequency dependence of the in-phase (χ'_M) susceptibility for **2** in zero DC field at various temperatures in the range 2.0 K (blue) to 50 K (red). Solid lines are a guide to the eye.

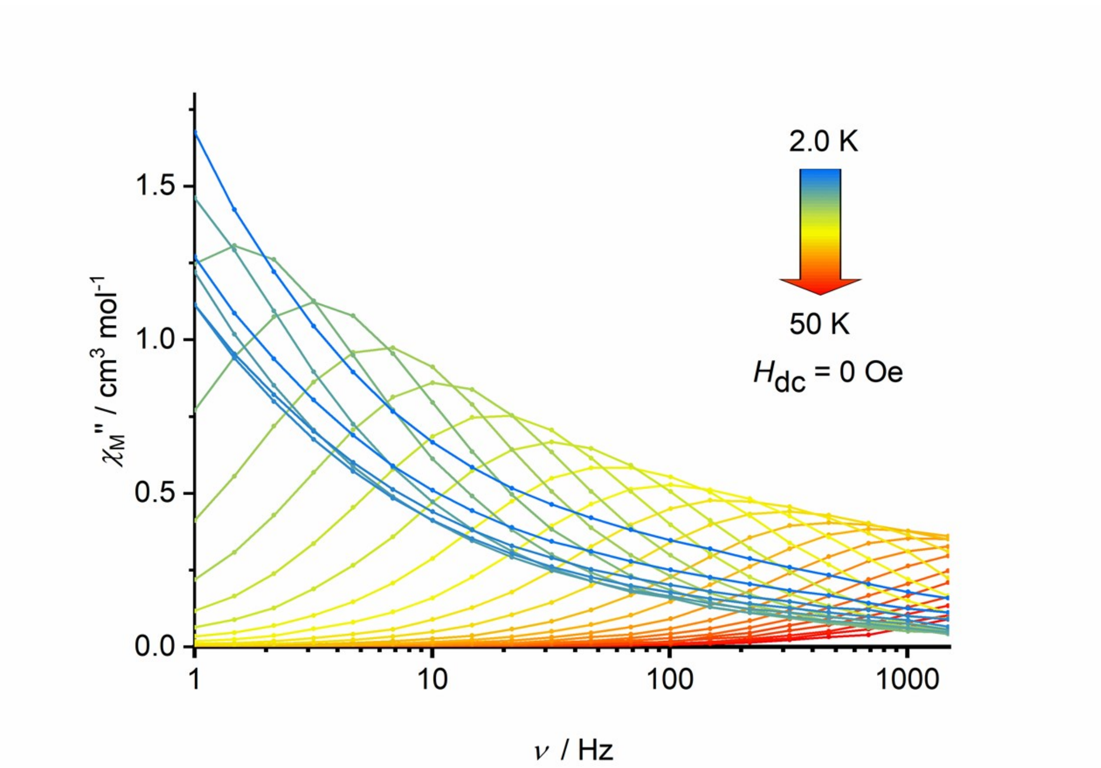

Fig. S9. Frequency dependence of the out-of-phase (χ''_M) susceptibility for **2** in zero DC field at various temperatures in the range 2.0 K (blue) to 50 K (red). Solid lines are a guide to the eye.

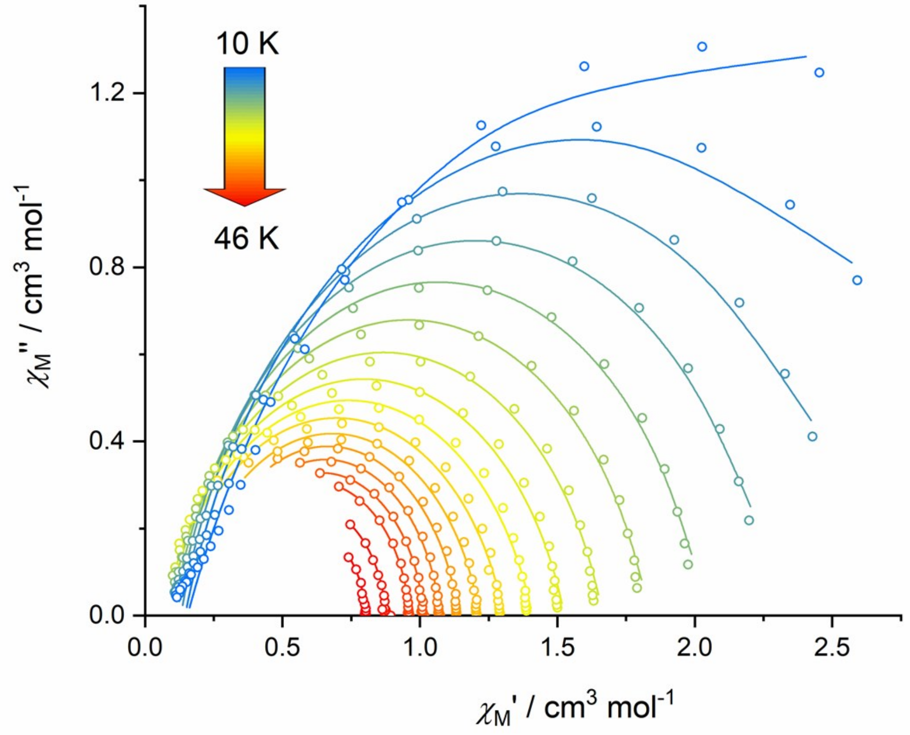

Fig. S10. Cole-Cole plots for the AC susceptibilities in zero DC field for **2** from 10-46 K. Solid lines represent fits to the data using equations 1 and 2, which describe χ' and χ'' in terms of frequency, isothermal susceptibility (χ_T), adiabatic susceptibility (χ_S), relaxation time (τ), and a variable representing the distribution of relaxation times (α).

$$\chi'(\nu_{ac}) = \chi_\infty + \frac{(\chi_s - \chi_\infty)[1 + (2\pi\nu_{ac}\tau)^{1-\alpha}\sin(\alpha\pi/2)]}{1 + 2(2\pi\nu_{ac}\tau)^{1-\alpha}\sin(\alpha\pi/2) + (2\pi\nu_{ac}\tau)^{2(1-\alpha)}} \qquad \text{Equation S1}$$

$$\chi''(\nu_{ac}) = \frac{(\chi_s - \chi_\infty)(2\pi\nu_{ac}\tau)^{1-\alpha}\cos(\alpha\pi/2)}{1 + 2(2\pi\nu_{ac}\tau)^{1-\alpha}\sin(\alpha\pi/2) + (2\pi\nu_{ac}\tau)^{2(1-\alpha)}} \qquad \text{Equation S2}$$

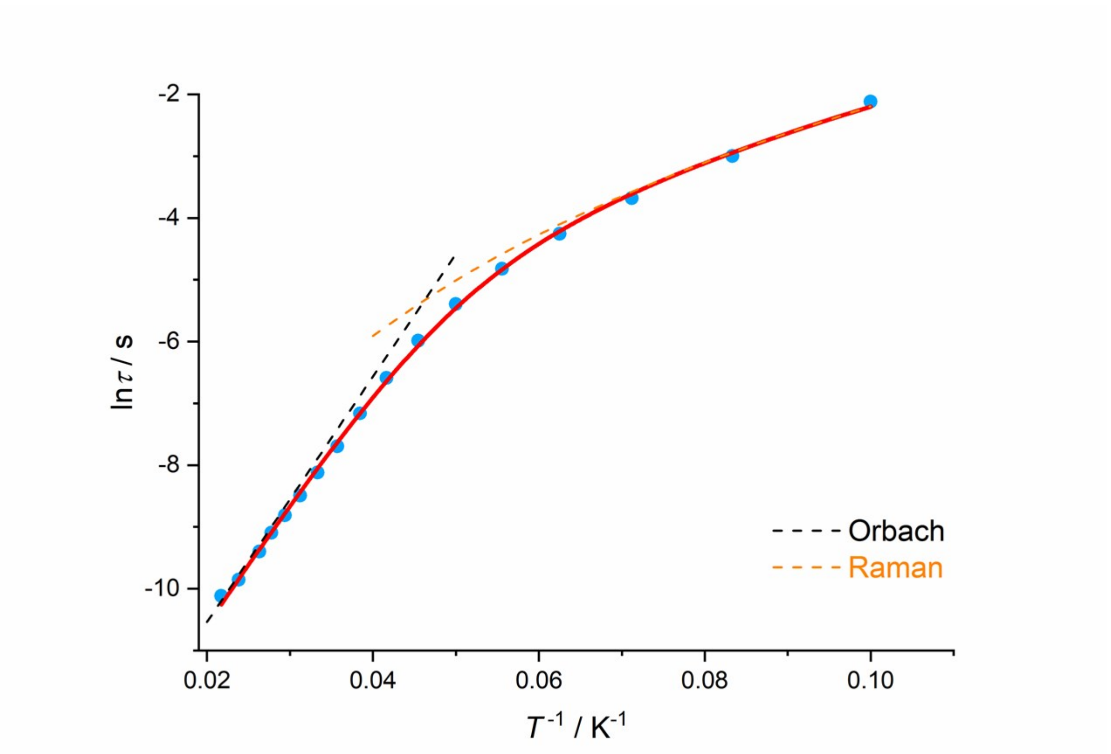

Fig. S11. Plot of natural log of the relaxation time (τ) *vs.* inverse temperature for **2**. The solid red line is the best fit (adjusted R^2 = 0.99937) to the equation $\tau^{-1} = \tau_0^{-1} e^{-U_{eff}/k_B T} + CT^n$, giving: U_{eff} = 138(4) cm^{-1}, τ_0 = 5.44(7) × 10^{-7} s, C = 8.03(4) × 10^{-4} s^{-1} K^{-n}, n = 4.05(2).

Table S4. Relaxation fitting parameters for **2** corresponding to Figs S10.

T / K	χ_T / cm^3 mol^{-1}	χ_S / cm^3 mol^{-1}	α	τ / s
46	0.80248(4.42193E-4)	0.38336(0.02524)	0.04023(0.01219)	4.03794E-5(3.23128E-6)
42	0.87604(0.00113)	0.31494(0.03978)	0.06413(0.01819)	5.25607E-5(5.22944E-6)
38	0.96177(9.10064E-4)	0.28151(0.01579)	0.06757(0.00854)	8.29553E-5(2.76076E-6)
36	1.01367(0.00109)	0.27233(0.01246)	0.07804(0.00763)	1.12475E-4(2.77815E-6)
34	1.07314(0.00145)	0.23862(0.012)	0.09638(0.0077)	1.49175E-4(3.28716E-6)
32	1.13962(0.0026)	0.19038(0.0159)	0.12611(0.0104)	2.04756E-4(5.64816E-6)
30	1.21678(0.00376)	0.14552(0.01699)	0.15614(0.0114)	2.97303E-4(8.4695E-6)
28	1.30477(0.0048)	0.08859(0.01615)	0.18361(0.01101)	4.55152E-4(1.19788E-5)
26	1.40833(0.00581)	0.07191(0.01389)	0.18907(0.01029)	7.75102E-4(1.76194E-5)
24	1.52557(0.00611)	0.06584(0.01052)	0.18541(0.0085)	0.00138(2.44678E-5)
22	1.66416(0.00616)	0.07285(0.00781)	0.17289(0.00684)	0.00251(3.43701E-5)
20	1.83046(0.00641)	0.08714(0.00612)	0.15716(0.0057)	0.00455(5.04053E-5)
18	2.03129(0.00625)	0.10296(0.00458)	0.14492(0.00442)	0.00807(6.83141E-5)
16	2.29451(0.00873)	0.11584(0.00483)	0.14722(0.0047)	0.01414(1.30245E-4)
14	2.63514(0.0152)	0.13043(0.006)	0.15823(0.00591)	0.02527(3.1423E-4)
12	3.14624(0.03193)	0.14542(0.00766)	0.18754(0.00784)	0.04974(0.00102)
10	4.06799(0.0951)	0.15412(0.00983)	0.25149(0.01106)	0.12033(0.00601)

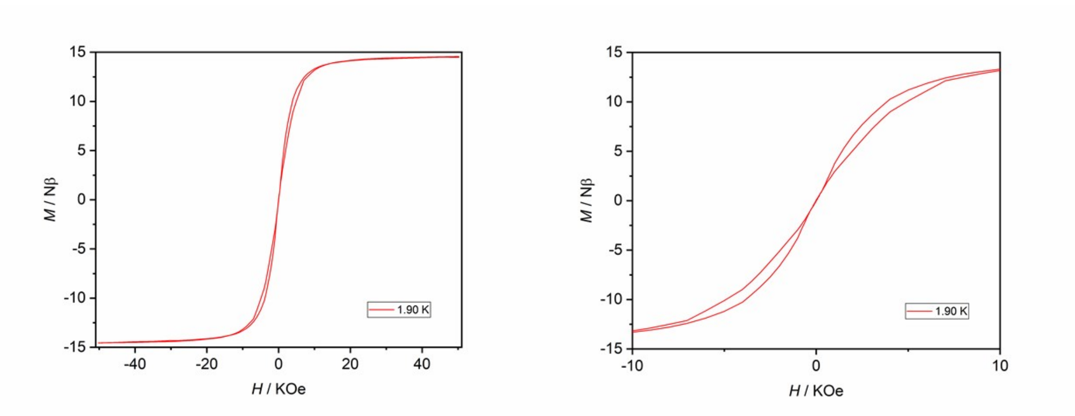

Fig. S12. Magnetic hysteresis loops for **2**. The data were collected at 1.9 K using an average field sweep rate of 27 Oe s^{-1}.

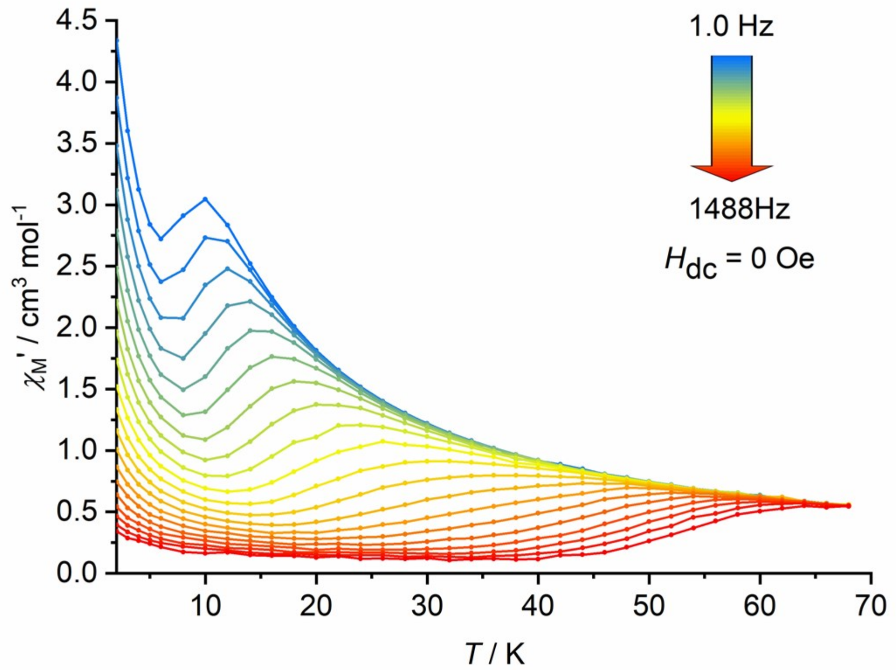

Fig. S13. Temperature dependence of the in-phase (χ'_M) AC susceptibility for [**3**][$B(C_6F_5)_4$] at various frequencies in the range 1.0 Hz (blue) to 1488 Hz (red) under zero DC field. Solid lines are a guide to the eye.

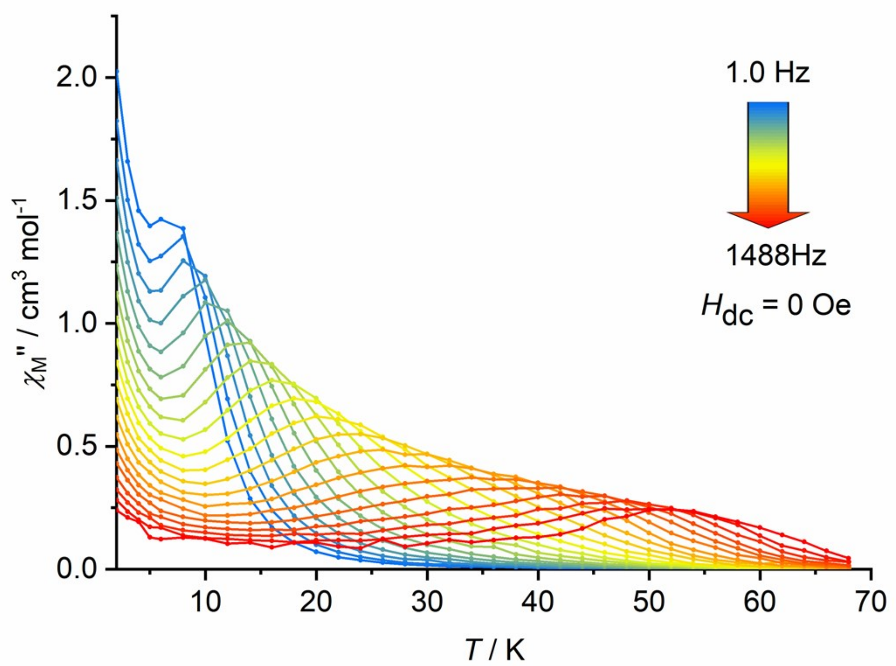

Fig. S14. Temperature dependence of the out-of-phase (χ''_M) AC susceptibility for [**3**][$B(C_6F_5)_4$] at various frequencies in the range 1.0 Hz (blue) to 1488 Hz (red) under zero DC field. Solid lines are a guide to the eye.

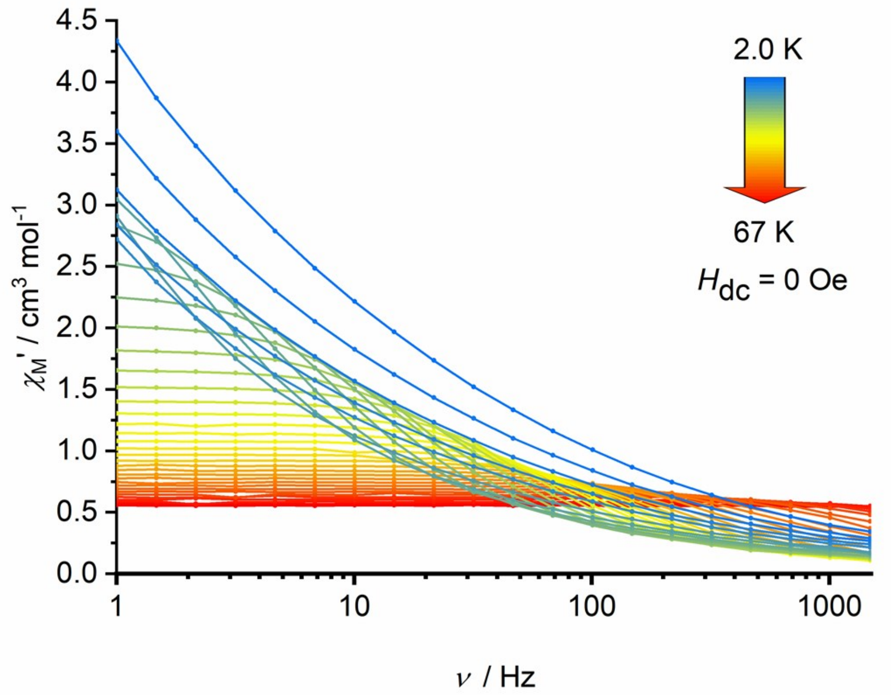

Fig. S15. Frequency dependence of the in-phase (χ'_M) susceptibility for [**3**][B(C_6F_5)$_4$] in zero DC field at various temperatures in the range 2 K (blue) to 67 K (red). Solid lines are a guide to the eye.

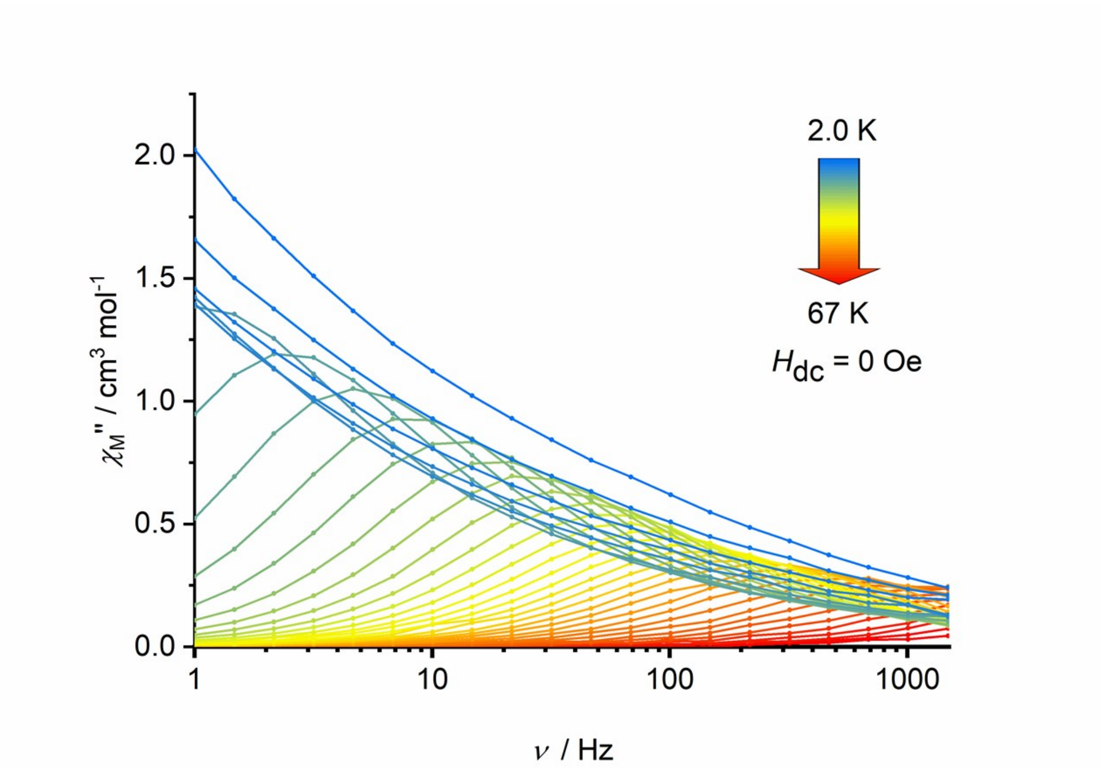

Fig. S16. Frequency dependence of the out-of-phase (χ''_M) susceptibility for [**3**][B(C_6F_5)$_4$] in zero DC field at various temperatures in the range 2 K (blue) to 67 K (red). Solid lines are a guide to the eye.

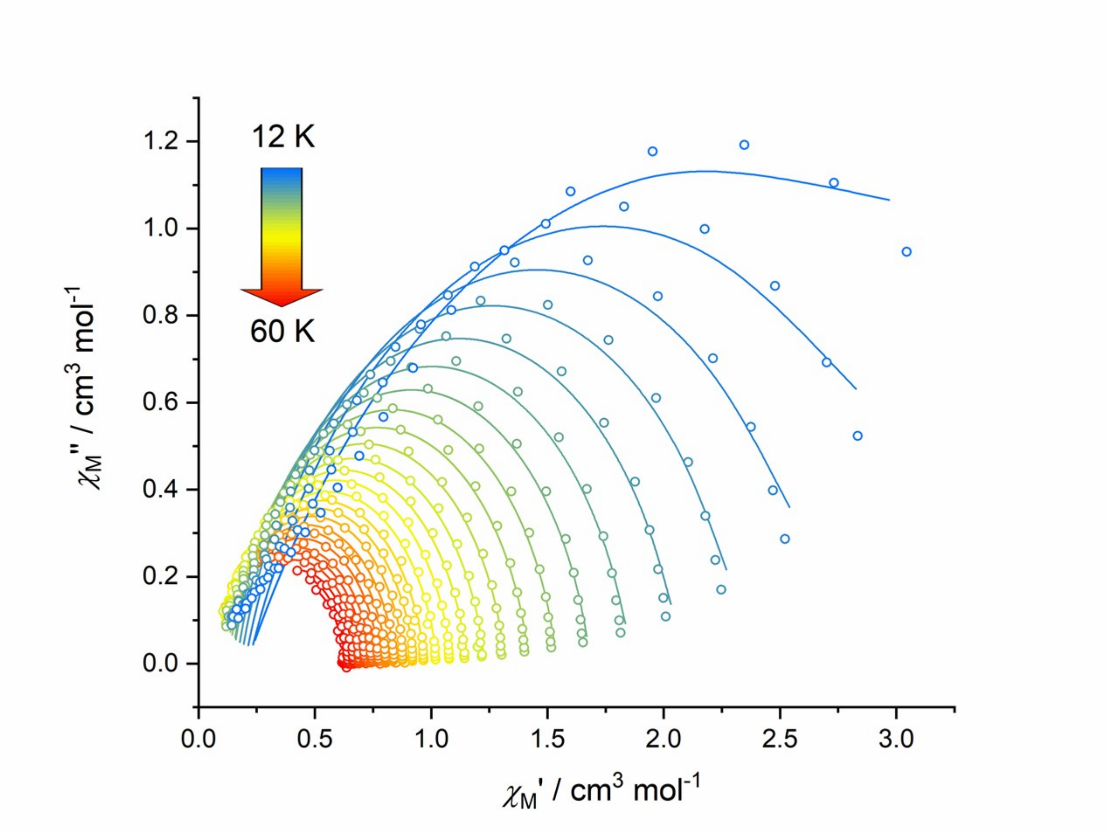

Fig. S17. Cole-Cole plots for the AC susceptibilities in zero DC field for [**3**][$B(C_6F_5)_4$] from 12-60 K. Solid lines represent fits to the data using equations S1 and S2.

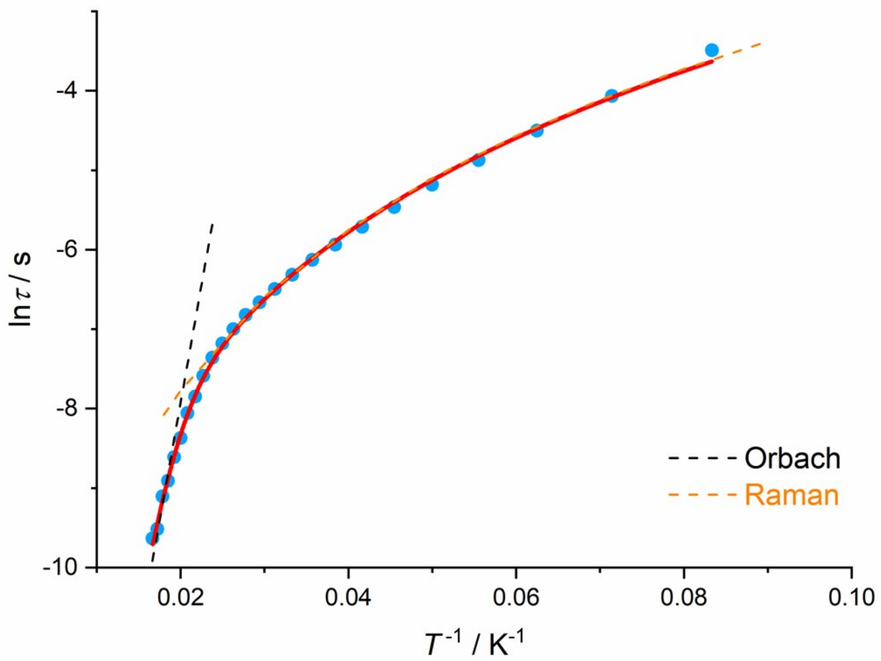

Fig. S18. Plot of natural log of the relaxation time (τ) *vs.* inverse temperature for [**3**][B(C_6F_5)$_4$]. The solid red line is the best fit (adjusted R^2 = 0.99912) to the equation $\tau^{-1} = \tau_0^{-1} e^{-U_{eff}/k_B T} + CT^n$, giving: U_{eff} = 411(23) cm^{-1}, τ_0 = 4.16(2) × 10^{-9} s, C = 2.66(3) × 10^{-4} s^{-1} K^{-n}, n = 2.92(1).

Table S5. Relaxation fitting parameters for [**3**][B(C_6F_5)$_4$] corresponding to Fig. S17.

T / K	χ_T / cm^3 mol^{-1}	χ_S / cm^3 mol^{-1}	α	τ / s
60	0.62435(0.00108)	0.22126(0.0269)	0.04743(0.02047)	6.54302E-5(6.00632E-6)
58	0.64414(0.00167)	0.16072(0.03435)	0.09222(0.02381)	7.36857E-5(7.82376E-6)
56	0.66786(8.72514E-4)	0.17756(0.0102)	0.08292(0.00934)	1.11018E-4(3.42453E-6)
54	0.69181(0.0011)	0.1348(0.01024)	0.09906(0.00927)	1.35223E-4(3.81848E-6)
52	0.71945(0.0015)	0.11415(0.01018)	0.11253(0.00993)	1.8203E-4(4.878E-6)
50	0.74704(0.00204)	0.0979(0.01109)	0.12433(0.01129)	2.31602E-4(6.55477E-6)
48	0.77848(0.00187)	0.09997(0.00758)	0.10456(0.00865)	3.16942E-4(5.86421E-6)
46	0.81142(0.00202)	0.07592(0.00707)	0.11684(0.00801)	3.90158E-4(6.54188E-6)
44	0.84688(0.00223)	0.08251(0.00649)	0.11476(0.00782)	5.07021E-4(7.85687E-6)
42	0.88513(0.00208)	0.08936(0.00519)	0.10258(0.00655)	6.36583E-4(7.83619E-6)
40	0.92737(0.0022)	0.07981(0.00496)	0.1082(0.00619)	7.59387E-4(8.79639E-6)
38	0.97318(0.00222)	0.07782(0.00454)	0.11201(0.00565)	9.12904E-4(9.60129E-6)
36	1.02584(0.00257)	0.08694(0.00476)	0.10946(0.00596)	0.00109(1.19299E-5)
34	1.0868(0.00223)	0.0878(0.00382)	0.10754(0.00469)	0.00128(1.09328E-5)
32	1.15418(0.00334)	0.0916(0.00528)	0.11277(0.00635)	0.00151(1.75964E-5)
30	1.22928(0.00377)	0.10443(0.00547)	0.11142(0.0065)	0.00181(2.1412E-5)
28	1.31774(0.00422)	0.10999(0.00561)	0.11256(0.0065)	0.00218(2.58516E-5)
26	1.42075(0.00577)	0.1189(0.00704)	0.11516(0.0079)	0.00264(3.80556E-5)
24	1.54063(0.00589)	0.12857(0.00651)	0.12069(0.00707)	0.00331(4.30091E-5)
22	1.68516(0.00812)	0.14265(0.00802)	0.12866(0.00841)	0.00424(6.64127E-5)
20	1.86118(0.01225)	0.14511(0.01064)	0.14386(0.01061)	0.00561(1.13603E-4)
18	2.0763(0.01512)	0.16371(0.01133)	0.15513(0.01083)	0.00765(1.61489E-4)
16	2.35489(0.02157)	0.18021(0.01334)	0.17482(0.01216)	0.01108(2.74384E-4)
14	2.72747(0.03494)	0.19845(0.01669)	0.20777(0.01448)	0.01715(5.55867E-4)
12	3.30624(0.0619)	0.21436(0.01979)	0.2629(0.01628)	0.03039(0.0014)

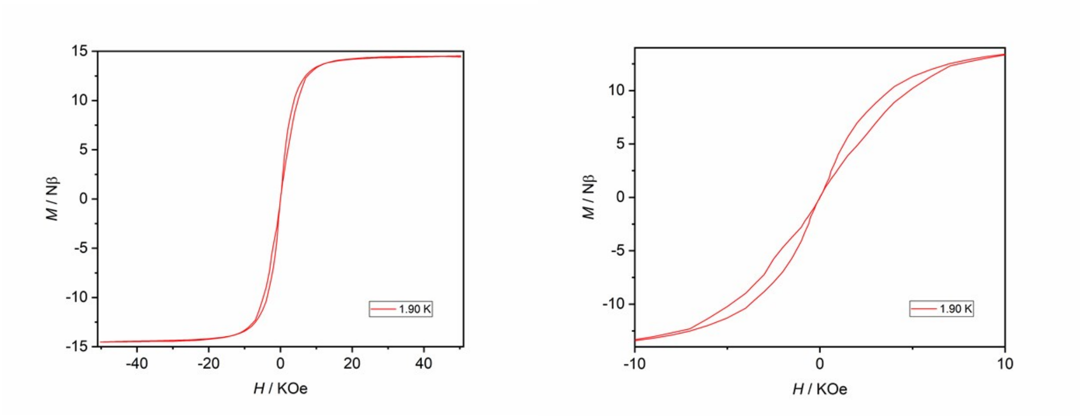

Fig. S19. Magnetic hysteresis loops for [**3**][B(C_6F_5)$_4$]. The data were collected at 1.9 K using an average field sweep rate of 29 Oe s^{-1}.

Computational details

The geometries of **2** and [**3**][B(C_6F_5)$_4$] were extracted from the crystal structure. Solvent molecules and the non-coordinated counter ion of [**3**][B(C_6F_5)$_4$] were removed from the structure. The positions of hydrogen atoms were optimized using density functional theory (DFT) while the positions of heavier atoms were kept frozen to their crystal-structure coordinates.

The geometry optimization was carried out using the *ADF2019* code version 1.03.[6] The pure GGA exchange-correlation functional PBE[7] was used along with the DFT-D3 dispersion correction[8] utilizing the Becke–Johnson damping function.[9] Scalar relativistic effects were treated with the zeroth-order regular approximation (ZORA).[10] All-electron Slater-type basis sets of triple-ζ quality with two sets of polarization functions (TZ2P) were used in the optimizations.[11] Static electron correlation effects were simulated by using fractional occupation numbers in the 4*f* orbitals. In practice this means that the two 4*f* β electrons of each Dy(III) ion were evenly distributed over the seven 4*f* orbitals giving a total of 21 β orbitals with an occupation of 0.2857. The "NumericalQuality" keyword in *ADF* was set to "Good" and the geometry convergence criteria were set to 10^{-4}, 10^{-4}, 10^{-3} and 10^{-1} atomic units for energy, gradient, bond distance and bond angles, respectively.

A set of multireference calculations were then carried out for each of the three ions in **2** and [**3**][B(C_6F_5)$_4$] using the OpenMolcas quantum chemistry software version 20.10.[12] The remaining ions were replaced by the diamagnetic Y(III) ion. The calculations were carried out using state-averaged complete active space self-consistent field (SA-CASSCF) approach.[13] The active space consisted of the seven 4*f* orbitals and the nine 4*f* electrons. All 21 sextet, 224 quartet and 490 doublets were solved for in three separate SA calculations. Spin-orbit coupling (SOC) was then introduced using the standard spin-orbit restricted active space state interaction (SO-RASSI) approach.[14] All 21 sextets and the lowest 128 quartets and 130 doublets corresponding to an energy cutoff of 50,000 cm^{-1} were included in the SO-RASSI treatment. The SOC operator was constructed using the atomic mean-field integral (AMFI) formalism[15] and diagonalized to yield the final spin-orbit coupled states. The local magnetic properties (**g**-tensors, crystal-field decomposition and effective local barrier) were calculated using the SINGLE_ANISO module.[16,17]

Relativistically contracted atomic natural orbital (ANO-RCC) basis sets were used in all multireference calculations.[18] A valence-polarized triple-ζ quality basis set was used for the Dy(III) ions whereas valence-polarized double-ζ quality basis sets were used for the remaining atoms. Scalar relativistic effects were introduced using the scalar exact two-component (X2C) transformation.[19] Integrals were stored using the Cholesky decomposition with a threshold of 10^{-8} atomic units.

The dipolar interations were calculated using the POLY_ANISO module.[17,20] The 16 lowest states of each Dy(III) ion corresponding to the ground J = 15/2 multiplet were used as an exchange basis. The reported exchange parameters correspond to the Ising-type pseudospin Hamiltonian

$$\tilde{H} = -J_{12}\tilde{S}_{z,1}\tilde{S}_{z,2} - J_{13}\tilde{S}_{z,1}\tilde{S}_{z,3} - J_{23}\tilde{S}_{z,2}\tilde{S}_{z,3} \quad (1)$$

where the indices 1, 2 and 3 correspond to the same indices of the Dy(III) ions used in the crystal structure. The operators $\tilde{S}_z$ act on the projection of pseudospin states that describe the local ground KD of each Dy(III) ion. The exchange parameters J_{12}, J_{13} and J_{23} were determined from the energy difference between the exchange eigenstates. Note that while we follow the usual practice and label the parameters J_{12}, J_{13} and J_{23} as exchange parameters and the resulting eigenstates as exchange eigenstates, the parameters describe dipolar coupling and not any exchange interaction.

Table S6. Properties of the eight lowest local KDs of the Dy1 ion of **2** corresponding to the crystal-field split states of the ground $^6H_{15/2}$ multiplet.

KD	E / cm^{-1}	g_x	g_y	g_z	θ [a]
KD1	0	0.00230	0.00346	19.59996	0.0°
KD2	185	0.02124	0.02125	16.98948	3.3°
KD3	359	0.08296	0.08664	14.66573	4.8°
KD4	457	1.91691	2.76384	11.55813	19.3°
KD5	487	0.47080	2.80944	14.58713	76.0°
KD6	534	4.85026	6.00605	9.37978	73.2°
KD7	606	1.06026	1.94509	15.75565	89.0°
KD8	748	0.04127	0.11175	19.49623	89.7°

[a] The angle between the principal magnetic axis of the given doublet and the that of the ground doublet.

Table S7. Properties of the eight lowest local KDs of the Dy2 ion of **2** corresponding to the crystal-field split states of the ground $^6H_{15/2}$ multiplet.

KD	E / cm^{-1}	g_x	g_y	g_z	θ [a]
KD1	0	0.00033	0.00050	19.71065	0.0°
KD2	190	0.00373	0.00434	17.33117	169.5°
KD3	330	0.01761	0.01928	15.08573	157.6°
KD4	446	0.16215	0.23800	11.64530	176.4°
KD5	524	1.76608	2.17045	8.66722	167.2°
KD6	561	3.02216	3.56685	13.96279	87.9°
KD7	584	1.06925	2.64913	13.51276	103.8°
KD8	659	0.23196	0.31895	18.65671	92.5°

[a] The angle between the principal magnetic axis of the given doublet and the that of the ground doublet.

Table S8. Properties of the eight lowest local KDs of the Dy3 ion of **2** corresponding to the crystal-field split states of the ground $^6H_{15/2}$ multiplet.

KD	E / cm^{-1}	g_x	g_y	g_z	θ [a]
KD1	0	0.00182	0.00354	19.45152	0.0°
KD2	151	0.01395	0.01440	16.96267	5.7°
KD3	338	0.29931	0.36689	14.61695	4.6°
KD4	427	2.71999	3.15657	14.71942	77.3°
KD5	456	1.09938	4.67216	9.44583	37.7°
KD6	510	4.83652	6.03930	10.34643	86.9°
KD7	575	0.52465	0.95643	15.87535	93.0°
KD8	732	0.06950	0.13148	19.54688	89.9°

[a] The angle between the principal magnetic axis of the given doublet and the that of the ground doublet.

Table S9. Properties of the eight lowest local KDs of the Dy1 ion of **3** corresponding to the crystal-field split states of the ground $^6H_{15/2}$ multiplet.

KD	E / cm^{-1}	g_x	g_y	g_z	θ ^{a}
KD1	0	0.00336	0.00518	19.76347	0.0°
KD2	324	0.15768	0.24916	16.61019	2.6°
KD3	495	2.20844	3.75104	12.17989	4.6°
KD4	577	6.97279	6.29181	4.92602	13.5°
KD5	663	0.19509	0.65238	11.15493	93.0°
KD6	765	0.20493	0.22674	13.80091	92.7°
KD7	901	0.04413	0.07052	16.64384	90.6°
KD8	1175	0.00149	0.00232	19.65495	89.4°

^{a} The angle between the principal magnetic axis of the given doublet and the that of the ground doublet.

Table S10. Properties of the eight lowest local KDs of the Dy2 ion of **3** corresponding to the crystal-field split states of the ground $^6H_{15/2}$ multiplet.

KD	E / cm^{-1}	g_x	g_y	g_z	θ ^{a}
KD1	0	0.00077	0.00082	19.83631	0.0°
KD2	285	0.02541	0.02669	17.08366	3.0°
KD3	453	0.18604	0.24426	14.51700	6.2°
KD4	557	0.49459	0.87408	11.73187	3.1°
KD5	643	3.27871	4.83529	8.39311	9.3°
KD6	727	7.99094	5.19896	0.57402	95.2°
KD7	776	2.11734	4.03301	14.80638	89.8°
KD8	855	0.12316	0.31070	18.60654	90.5°

^{a} The angle between the principal magnetic axis of the given doublet and the that of the ground doublet.

Table S11. Properties of the eight lowest local KDs of the Dy3 ion of **3** corresponding to the crystal-field split states of the ground $^6H_{15/2}$ multiplet.

KD	E / cm^{-1}	g_x	g_y	g_z	θ ^{a}
KD1	0	0.00291	0.00443	19.77139	0.0°
KD2	337	0.15944	0.25628	16.56737	1.6°
KD3	506	2.19075	3.64597	12.17660	2.3°
KD4	590	5.35063	5.82131	6.89882	94.8°
KD5	677	0.88917	1.05879	11.12947	90.5°
KD6	784	0.11243	0.12730	13.77847	89.2°
KD7	929	0.01332	0.02057	16.62206	89.1°
KD8	1210	0.00055	0.00093	19.64265	89.9°

^{a} The angle between the principal magnetic axis of the given doublet and the that of the ground doublet.

Table S12. Local *ab initio* CF parameters (in cm^{-1}) calculated for the Dy1 ion of **2** given in the Iwahara–Chibotaru notation.[21]

k	*q*	Re(B_{kq})	Im(B_{kq})	$\lvert B_{kq}\rvert$
2	0	−381.122903	0.000000	381.122903
2	1	−8.397474	−4.008712	9.305232
2	2	89.218927	3.856960	89.302257
4	0	−24.498418	−0.000000	24.498418
4	1	−0.693608	8.502927	8.531170
4	2	6.831846	2.028612	7.126667
4	3	−3.970945	1.796030	4.358225
4	4	12.122580	−1.521283	12.217661
6	0	2.725866	0.000000	2.725866
6	1	2.361576	−6.681363	7.086441
6	2	27.625550	0.600328	27.632072
6	3	−0.017302	−4.853108	4.853139
6	4	1.769209	5.179451	5.473282
6	5	−3.426823	0.367473	3.446469
6	6	−3.780842	−2.540088	4.554866
8	0	0.176798	−0.000000	0.176798
8	1	−0.038223	0.082721	0.091125
8	2	−0.668237	−0.011222	0.668331
8	3	−0.005825	0.168331	0.168431
8	4	−0.092627	−0.066038	0.113758
8	5	0.029500	−0.008923	0.030820
8	6	0.016997	0.023669	0.029140
8	7	−0.008999	−0.000962	0.009050
8	8	0.000150	−0.001158	0.001168
10	0	0.018217	0.000000	0.018217
10	1	−0.002270	0.026767	0.026863
10	2	−0.018630	0.002251	0.018765
10	3	−0.005357	0.008941	0.010423
10	4	−0.003670	−0.003350	0.004969
10	5	0.001002	0.002198	0.002415
10	6	0.001198	0.003270	0.003483
10	7	−0.004552	0.000114	0.004554
10	8	−0.002724	−0.001911	0.003327
10	9	−0.000904	0.001117	0.001437
10	10	−0.001832	−0.001400	0.002305
12	0	0.007336	0.000000	0.007336
12	1	−0.000812	−0.004229	0.004307
12	2	0.000561	0.000836	0.001007
12	3	0.000264	−0.000802	0.000844
12	4	0.001844	0.000210	0.001856
12	5	−0.000171	−0.000399	0.000434

12	6	0.000062	0.000111	0.000127
12	7	0.000196	−0.000099	0.000220
12	8	0.000041	0.000139	0.000145
12	9	−0.000021	−0.000063	0.000066
12	10	0.000063	−0.000047	0.000079
12	11	0.000057	0.000029	0.000064
12	12	0.000021	0.000059	0.000063
14	0	−0.000017	0.000000	0.000017
14	1	0.000000	0.000002	0.000003
14	2	−0.000002	−0.000009	0.000009
14	3	−0.000000	0.000002	0.000002
14	4	−0.000003	0.000003	0.000005
14	5	−0.000002	0.000002	0.000003
14	6	−0.000001	−0.000003	0.000004
14	7	0.000000	−0.000001	0.000001
14	8	0.000001	−0.000001	0.000001
14	9	0.000000	−0.000000	0.000000
14	10	−0.000000	−0.000000	0.000000
14	11	0.000000	0.000000	0.000000
14	12	−0.000000	−0.000000	0.000000
14	13	0.000000	0.000000	0.000000
14	14	0.000000	0.000000	0.000000

[a] The CF parameters are only listed for non-negative values of q. The values with negative q are given by B_{k-q} = $(-1)^q B_{kq}*$.

Table S13. Local *ab initio* CF parameters (in cm^{-1}) calculated for the Dy2 ion of **2** given in the Iwahara–Chibotaru notation.[21]

k	q	Re(B_{kq})	Im(B_{kq})	$\lvert B_{kq}\rvert$
2	0	−371.141373	−0.000000	371.141373
2	1	−43.892471	−10.439426	45.116855
2	2	22.941625	−11.584792	25.700691
4	0	−29.013705	0.000000	29.013705
4	1	8.673500	1.816138	8.861601
4	2	6.569137	4.706265	8.080996
4	3	7.553213	2.878312	8.083050
4	4	0.750967	−1.066991	1.304768
6	0	−0.301283	−0.000000	0.301283
6	1	8.338964	2.773080	8.787963
6	2	24.045289	10.125736	26.090352
6	3	−1.720624	−0.711764	1.862030
6	4	−3.007725	−2.219296	3.737872
6	5	1.357193	2.837822	3.145665
6	6	−2.840887	−2.178229	3.579849
8	0	0.111594	−0.000000	0.111594
8	1	−0.117670	−0.048705	0.127351
8	2	−0.516388	−0.221703	0.561969
8	3	−0.003179	0.004416	0.005441
8	4	0.025041	0.036566	0.044318
8	5	−0.018854	−0.029428	0.034950
8	6	0.003895	−0.002776	0.004783
8	7	0.002127	0.006258	0.006609
8	8	0.001116	0.005243	0.005360
10	0	0.004081	−0.000000	0.004081
10	1	−0.012774	−0.006102	0.014157
10	2	−0.031312	−0.014478	0.034497
10	3	0.004594	0.004593	0.006496
10	4	−0.002152	−0.000152	0.002157
10	5	0.000801	−0.001010	0.001289
10	6	0.000086	0.000642	0.000648
10	7	−0.000954	0.000816	0.001256
10	8	−0.001715	−0.002423	0.002969
10	9	−0.000173	−0.000214	0.000275
10	10	−0.000001	0.000189	0.000189
12	0	0.005817	0.000000	0.005817
12	1	−0.004023	−0.000115	0.004025
12	2	−0.000312	0.000703	0.000769
12	3	0.000781	0.000440	0.000897
12	4	0.000965	0.000874	0.001302
12	5	−0.000055	0.000026	0.000061

12	6	−0.000038	−0.000271	0.000274
12	7	0.000069	0.000099	0.000120
12	8	0.000050	0.000087	0.000101
12	9	0.000012	−0.000042	0.000044
12	10	−0.000001	0.000055	0.000055
12	11	0.000010	−0.000044	0.000045
12	12	0.000011	0.000038	0.000040
14	0	−0.000017	−0.000000	0.000017
14	1	0.000000	−0.000001	0.000001
14	2	0.000002	0.000002	0.000002
14	3	−0.000005	−0.000006	0.000007
14	4	−0.000000	−0.000000	0.000000
14	5	−0.000000	0.000001	0.000001
14	6	0.000000	0.000002	0.000002
14	7	−0.000001	−0.000001	0.000001
14	8	0.000000	0.000000	0.000000
14	9	−0.000000	−0.000000	0.000000
14	10	−0.000000	−0.000000	0.000000
14	11	−0.000000	0.000000	0.000000
14	12	0.000000	−0.000000	0.000000
14	13	0.000000	0.000000	0.000000
14	14	−0.000000	0.000000	0.000000

[a] The CF parameters are only listed for non-negative values of q. The values with negative q are given by $B_{k-q} = (-1)^q B_{kq}*$.

Table S14. Local *ab initio* CF parameters (in cm^{-1}) calculated for the Dy3 ion of **2** given in the Iwahara–Chibotaru notation.[21]

k	*q*	Re(B_{kq})	Im(B_{kq})	$\|B_{kq}\|$
2	0	−361.310776	−0.000000	361.310776
2	1	6.833554	−2.072891	7.141032
2	2	102.032093	20.071583	103.987578
4	0	−22.662639	0.000000	22.662639
4	1	5.187683	−1.834103	5.502362
4	2	5.761216	3.310805	6.644775
4	3	5.459589	14.679976	15.662337
4	4	−2.355664	14.592363	14.781279
6	0	11.923919	−0.000000	11.923919
6	1	−4.006854	8.604641	9.491824
6	2	28.559318	8.896350	29.912869
6	3	−2.107148	7.548798	7.837373
6	4	−7.012843	−0.846296	7.063723
6	5	4.515018	5.271474	6.940737
6	6	1.133209	−2.054734	2.346507
8	0	0.170292	0.000000	0.170292
8	1	0.073508	−0.157657	0.173952
8	2	−0.590168	−0.241895	0.637817
8	3	0.126865	−0.241402	0.272708
8	4	0.018341	−0.034522	0.039091
8	5	−0.046157	−0.060869	0.076390
8	6	−0.016669	−0.001835	0.016770
8	7	0.011588	0.018863	0.022139
8	8	−0.009400	0.009004	0.013017
10	0	0.007509	−0.000000	0.007509
10	1	0.005309	−0.016280	0.017124
10	2	−0.034364	−0.000611	0.034369
10	3	0.007069	0.000742	0.007108
10	4	−0.000458	0.001642	0.001705
10	5	0.003873	0.001973	0.004346
10	6	−0.006719	0.000211	0.006722
10	7	0.000709	0.003741	0.003808
10	8	0.000554	−0.001490	0.001589
10	9	−0.000805	0.002257	0.002396
10	10	0.001030	0.000395	0.001103
12	0	0.008057	0.000000	0.008057
12	1	−0.000036	0.007164	0.007164
12	2	0.000513	0.001755	0.001828
12	3	−0.000605	0.000772	0.000981
12	4	0.001499	0.001307	0.001989
12	5	−0.000510	0.001158	0.001265

12	6	0.000068	−0.000306	0.000313
12	7	0.000001	−0.000242	0.000242
12	8	−0.000035	0.000121	0.000126
12	9	−0.000088	−0.000190	0.000210
12	10	−0.000052	0.000161	0.000170
12	11	0.000054	−0.000019	0.000057
12	12	−0.000006	−0.000018	0.000019
14	0	−0.000005	0.000000	0.000005
14	1	0.000001	−0.000001	0.000002
14	2	0.000004	−0.000004	0.000006
14	3	−0.000001	−0.000011	0.000011
14	4	−0.000012	−0.000002	0.000012
14	5	0.000005	0.000000	0.000005
14	6	0.000005	0.000001	0.000005
14	7	−0.000003	−0.000001	0.000004
14	8	0.000001	0.000001	0.000001
14	9	−0.000001	−0.000000	0.000001
14	10	0.000001	−0.000000	0.000001
14	11	−0.000000	0.000000	0.000000
14	12	−0.000000	0.000000	0.000000
14	13	0.000000	0.000000	0.000000
14	14	−0.000000	−0.000000	0.000000

[a] The CF parameters are only listed for non-negative values of q. The values with negative q are given by $B_{k-q} = (-1)^q B_{kq}^*$.

Table S15. Local *ab initio* CF parameters (in cm^{-1}) calculated for the Dy1 ion of **3** given in the Iwahara–Chibotaru notation.[21]

k	*q*	Re(B_{kq})	Im(B_{kq})	$\|B_{kq}\|$
2	0	−537.587247	−0.000000	537.587247
2	1	5.472878	−1.345256	5.635788
2	2	208.153409	11.929195	208.494957
4	0	−36.495770	−0.000000	36.495770
4	1	4.741346	−0.557716	4.774034
4	2	−9.902624	−0.628474	9.922547
4	3	1.873363	4.236424	4.632146
4	4	−2.893290	−1.159578	3.117009
6	0	−27.765751	−0.000000	27.765751
6	1	−4.513450	1.836708	4.872856
6	2	19.239634	−4.350506	19.725375
6	3	1.304738	0.600621	1.436345
6	4	−2.366571	1.794675	2.970104
6	5	1.196963	−1.418767	1.856239
6	6	9.056830	2.061586	9.288503
8	0	1.030300	0.000000	1.030300
8	1	0.228393	−0.026387	0.229913
8	2	−0.736197	0.107085	0.743945
8	3	0.050198	−0.045127	0.067500
8	4	−0.070601	−0.019210	0.073168
8	5	−0.010823	0.016161	0.019450
8	6	−0.033307	−0.005996	0.033842
8	7	0.008687	−0.006296	0.010728
8	8	0.028407	0.011165	0.030522
10	0	0.019098	−0.000000	0.019098
10	1	−0.012827	−0.001453	0.012909
10	2	−0.017867	0.007812	0.019500
10	3	−0.001251	−0.004921	0.005078
10	4	0.001998	0.000814	0.002158
10	5	−0.001115	0.002007	0.002296
10	6	−0.007835	−0.000345	0.007843
10	7	−0.001537	−0.000552	0.001634
10	8	0.006041	−0.000274	0.006047
10	9	−0.000018	0.001333	0.001333
10	10	−0.001803	−0.000596	0.001898
12	0	0.005482	−0.000000	0.005482
12	1	0.002866	0.000001	0.002866
12	2	−0.003829	0.000818	0.003915
12	3	−0.000294	−0.000006	0.000294
12	4	0.001242	−0.000523	0.001347
12	5	0.000058	0.000209	0.000217

12	6	−0.000162	−0.000100	0.000190
12	7	0.000138	−0.000039	0.000143
12	8	−0.000108	0.000005	0.000108
12	9	0.000023	0.000027	0.000036
12	10	−0.000125	0.000013	0.000126
12	11	0.000051	−0.000043	0.000067
12	12	0.000240	0.000108	0.000263
14	0	−0.000024	−0.000000	0.000024
14	1	−0.000021	−0.000001	0.000021
14	2	0.000017	−0.000002	0.000017
14	3	−0.000007	0.000001	0.000007
14	4	−0.000001	−0.000001	0.000001
14	5	0.000000	−0.000001	0.000001
14	6	0.000005	0.000001	0.000005
14	7	0.000001	−0.000000	0.000001
14	8	−0.000001	−0.000000	0.000001
14	9	0.000000	−0.000000	0.000000
14	10	0.000001	0.000000	0.000001
14	11	−0.000000	0.000000	0.000000
14	12	−0.000001	−0.000000	0.000001
14	13	−0.000000	−0.000000	0.000000
14	14	0.000000	0.000000	0.000000

[a] The CF parameters are only listed for non-negative values of q. The values with negative q are given by $B_{k-q} = (-1)^q B_{kq}{}^*$.

Table S16. Local *ab initio* CF parameters (in cm^{-1}) calculated for the Dy2 ion of **3** given in the Iwahara–Chibotaru notation.[21]

k	q	Re(B_{kq})	Im(B_{kq})	$\|B_{kq}\|$
2	0	−483.210074	0.000000	483.210074
2	1	3.343657	9.966012	10.511966
2	2	2.107003	23.706664	23.800113
4	0	−31.202690	−0.000000	31.202690
4	1	0.038882	0.261197	0.264075
4	2	−2.441003	6.539380	6.980114
4	3	4.866046	−0.820107	4.934671
4	4	−14.600859	−12.470603	19.201589
6	0	−18.049171	−0.000000	18.049171
6	1	−0.842077	−3.735097	3.828843
6	2	−6.248396	17.365289	18.455235
6	3	−2.158904	−0.069773	2.160032
6	4	−1.511920	−1.216984	1.940863
6	5	0.127485	−1.307383	1.313584
6	6	−7.965113	4.349678	9.075391
8	0	0.336488	−0.000000	0.336488
8	1	0.012448	0.110116	0.110817
8	2	0.187704	−0.539755	0.571461
8	3	0.054464	−0.008637	0.055144
8	4	−0.001787	0.018595	0.018681
8	5	−0.002025	0.015835	0.015964
8	6	0.025489	−0.020341	0.032611
8	7	−0.002839	−0.003979	0.004888
8	8	−0.001384	0.005732	0.005897
10	0	0.027343	0.000000	0.027343
10	1	−0.002173	0.000958	0.002375
10	2	0.008722	−0.017292	0.019367
10	3	−0.002209	0.003456	0.004102
10	4	0.007971	0.003767	0.008816
10	5	0.000008	0.001410	0.001410
10	6	0.005368	−0.002174	0.005791
10	7	0.001430	0.001326	0.001950
10	8	−0.000800	−0.005452	0.005510
10	9	−0.000745	0.000805	0.001097
10	10	0.006422	0.001353	0.006563
12	0	0.004274	−0.000000	0.004274
12	1	0.001305	0.001441	0.001944
12	2	−0.000126	−0.002286	0.002290
12	3	0.000266	−0.000035	0.000269
12	4	−0.000398	−0.000453	0.000603
12	5	0.000156	−0.000028	0.000158

12	6	0.000084	−0.000260	0.000273
12	7	−0.000070	−0.000091	0.000114
12	8	0.000021	0.000172	0.000173
12	9	0.000009	−0.000000	0.000009
12	10	0.000025	0.000001	0.000025
12	11	0.000019	0.000044	0.000048
12	12	0.000134	−0.000206	0.000246
14	0	−0.000010	0.000000	0.000010
14	1	−0.000003	−0.000003	0.000004
14	2	0.000001	0.000004	0.000004
14	3	0.000001	−0.000001	0.000001
14	4	−0.000003	−0.000002	0.000003
14	5	−0.000000	−0.000001	0.000001
14	6	−0.000003	0.000003	0.000004
14	7	0.000000	−0.000000	0.000000
14	8	0.000001	0.000001	0.000001
14	9	−0.000000	−0.000000	0.000000
14	10	−0.000000	0.000000	0.000000
14	11	−0.000000	−0.000000	0.000000
14	12	−0.000000	0.000001	0.000001
14	13	−0.000000	−0.000000	0.000000
14	14	0.000000	0.000000	0.000000

[a] The CF parameters are only listed for non-negative values of q. The values with negative q are given by $B_{k-q} = (-1)^q B_{kq}*$.

Table S17. Local *ab initio* CF parameters (in cm^{-1}) calculated for the Dy3 ion of **3** given in the Iwahara–Chibotaru notation.[21]

k	*q*	Re(B_{kq})	Im(B_{kq})	$\|B_{kq}\|$
2	0	−553.033767	−0.000000	553.033767
2	1	3.644463	−0.042657	3.644712
2	2	215.826710	−3.399823	215.853486
4	0	−34.184053	−0.000000	34.184053
4	1	−2.630526	−0.996235	2.812855
4	2	−9.950848	−1.024055	10.003403
4	3	−3.465691	4.045340	5.326893
4	4	−2.688455	0.415055	2.720306
6	0	−31.821191	0.000000	31.821191
6	1	−0.050318	1.340573	1.341517
6	2	18.379368	−0.593269	18.388940
6	3	−1.003343	0.075727	1.006197
6	4	−1.422972	−0.055768	1.424064
6	5	−2.966653	−1.504834	3.326493
6	6	8.927169	0.785113	8.961626
8	0	1.111640	−0.000000	1.111640
8	1	0.000045	−0.026861	0.026861
8	2	−0.745261	0.021811	0.745580
8	3	0.016299	−0.026630	0.031222
8	4	−0.087208	0.008314	0.087603
8	5	0.035276	0.016998	0.039158
8	6	−0.034145	−0.004984	0.034507
8	7	−0.012321	−0.007200	0.014270
8	8	0.031965	0.002350	0.032051
10	0	0.017995	0.000000	0.017995
10	1	0.004663	−0.001314	0.004845
10	2	−0.012962	0.001463	0.013044
10	3	0.002275	−0.003559	0.004224
10	4	0.002015	−0.000034	0.002015
10	5	0.003353	0.002431	0.004142
10	6	−0.007927	−0.000837	0.007971
10	7	−0.000911	−0.000089	0.000916
10	8	0.005264	−0.000081	0.005265
10	9	−0.000389	0.001012	0.001085
10	10	−0.001736	−0.000161	0.001743
12	0	0.005958	0.000000	0.005958
12	1	−0.000069	0.000026	0.000074
12	2	−0.004131	0.000070	0.004131
12	3	0.000065	−0.000026	0.000070
12	4	0.000994	−0.000054	0.000995
12	5	0.000097	0.000148	0.000177

12	6	−0.000196	−0.000006	0.000196
12	7	−0.000023	−0.000064	0.000068
12	8	−0.000069	0.000003	0.000070
12	9	0.000020	0.000005	0.000021
12	10	−0.000076	0.000015	0.000077
12	11	−0.000097	−0.000062	0.000115
12	12	0.000240	0.000044	0.000244
14	0	−0.000031	−0.000000	0.000031
14	1	0.000002	−0.000001	0.000002
14	2	0.000020	−0.000000	0.000020
14	3	0.000000	0.000002	0.000002
14	4	0.000001	−0.000001	0.000001
14	5	−0.000002	−0.000001	0.000002
14	6	0.000005	0.000001	0.000005
14	7	0.000000	−0.000000	0.000000
14	8	−0.000001	0.000000	0.000001
14	9	−0.000000	−0.000000	0.000000
14	10	0.000001	−0.000000	0.000001
14	11	0.000000	0.000000	0.000001
14	12	−0.000001	−0.000000	0.000001
14	13	−0.000000	−0.000000	0.000000
14	14	0.000000	0.000000	0.000000

[a] The CF parameters are only listed for non-negative values of q. The values with negative q are given by $B_{k-q} = (-1)^q B_{kq}{}^*$.

Table S18. Squared magnitudes of projections of the local *ab initio* CF eigenstates calculated for the Dy1 ion of **2** onto pseudospin eigenstates with pseudospin J = 15/2 and projection M.

M	KD1		KD2		KD3		KD4		KD5		KD6		KD7		KD8	
−15/2	0.013	0.934	0.000	0.000	0.016	0.035	0.000	0.000	0.000	0.000	0.001	0.000	0.000	0.000	0.000	0.000
−13/2	0.000	0.000	0.764	0.197	0.001	0.003	0.007	0.022	0.002	0.003	0.001	0.000	0.001	0.000	0.000	0.000
−11/2	0.001	0.052	0.003	0.001	0.286	0.633	0.003	0.005	0.002	0.000	0.009	0.001	0.004	0.000	0.000	0.000
−9/2	0.000	0.000	0.027	0.007	0.004	0.007	0.220	0.613	0.043	0.050	0.010	0.002	0.006	0.009	0.001	0.001
−7/2	0.000	0.000	0.000	0.000	0.003	0.008	0.020	0.026	0.114	0.077	0.590	0.013	0.078	0.053	0.016	0.002
−5/2	0.000	0.000	0.001	0.000	0.000	0.000	0.019	0.015	0.112	0.078	0.062	0.089	0.179	0.319	0.004	0.121
−3/2	0.000	0.000	0.000	0.000	0.001	0.001	0.025	0.011	0.091	0.143	0.013	0.061	0.221	0.089	0.328	0.016
−1/2	0.000	0.000	0.000	0.000	0.001	0.001	0.009	0.005	0.181	0.103	0.112	0.036	0.011	0.030	0.029	0.481
1/2	0.000	0.000	0.000	0.000	0.001	0.001	0.005	0.009	0.103	0.181	0.036	0.112	0.030	0.011	0.481	0.029
3/2	0.000	0.000	0.000	0.000	0.001	0.001	0.011	0.025	0.143	0.091	0.061	0.013	0.089	0.221	0.016	0.328
5/2	0.000	0.000	0.000	0.001	0.000	0.000	0.015	0.019	0.078	0.112	0.089	0.062	0.319	0.179	0.121	0.004
7/2	0.000	0.000	0.000	0.000	0.008	0.003	0.026	0.020	0.077	0.114	0.013	0.590	0.053	0.078	0.002	0.016
9/2	0.000	0.000	0.007	0.027	0.007	0.004	0.613	0.220	0.050	0.043	0.002	0.010	0.009	0.006	0.001	0.001
11/2	0.052	0.001	0.001	0.003	0.633	0.286	0.005	0.003	0.000	0.002	0.001	0.009	0.000	0.004	0.000	0.000
13/2	0.000	0.000	0.197	0.764	0.003	0.001	0.022	0.007	0.003	0.002	0.000	0.001	0.000	0.001	0.000	0.000
15/2	0.934	0.013	0.000	0.000	0.035	0.016	0.000	0.000	0.000	0.000	0.000	0.001	0.000	0.000	0.000	0.000

Table S19. Squared magnitudes of projections of the local *ab initio* CF eigenstates calculated for the Dy2 ion of **2** onto pseudospin eigenstates with pseudospin J = 15/2 and projection M.

M	KD1		KD2		KD3		KD4		KD5		KD6		KD7		KD8	
−15/2	0.082	0.885	0.002	0.000	0.001	0.026	0.003	0.002	0.000	0.000	0.000	0.000	0.000	0.000	0.000	0.000
−13/2	0.000	0.000	0.826	0.128	0.001	0.025	0.011	0.007	0.000	0.001	0.000	0.000	0.001	0.000	0.000	0.000
−11/2	0.003	0.030	0.035	0.005	0.018	0.690	0.127	0.080	0.001	0.004	0.000	0.001	0.004	0.000	0.000	0.002
−9/2	0.000	0.000	0.003	0.001	0.005	0.198	0.330	0.210	0.054	0.170	0.006	0.003	0.013	0.000	0.001	0.008
−7/2	0.000	0.000	0.000	0.000	0.001	0.032	0.090	0.055	0.097	0.465	0.032	0.166	0.051	0.002	0.003	0.006
−5/2	0.000	0.000	0.000	0.000	0.000	0.003	0.043	0.034	0.050	0.035	0.107	0.134	0.406	0.114	0.008	0.066
−3/2	0.000	0.000	0.000	0.000	0.000	0.000	0.001	0.003	0.009	0.073	0.168	0.097	0.191	0.101	0.238	0.119
−1/2	0.000	0.000	0.000	0.000	0.000	0.001	0.001	0.004	0.038	0.005	0.127	0.159	0.006	0.111	0.105	0.445
1/2	0.000	0.000	0.000	0.000	0.001	0.000	0.004	0.001	0.005	0.038	0.159	0.127	0.111	0.006	0.445	0.105
3/2	0.000	0.000	0.000	0.000	0.000	0.000	0.003	0.001	0.073	0.009	0.097	0.168	0.101	0.191	0.119	0.238
5/2	0.000	0.000	0.000	0.000	0.003	0.000	0.034	0.043	0.035	0.050	0.134	0.107	0.114	0.406	0.066	0.008
7/2	0.000	0.000	0.000	0.000	0.032	0.001	0.055	0.090	0.465	0.097	0.166	0.032	0.002	0.051	0.006	0.003
9/2	0.000	0.000	0.001	0.003	0.198	0.005	0.210	0.330	0.170	0.054	0.003	0.006	0.000	0.013	0.008	0.001
11/2	0.030	0.003	0.005	0.035	0.690	0.018	0.080	0.127	0.004	0.001	0.001	0.000	0.000	0.004	0.002	0.000
13/2	0.000	0.000	0.128	0.826	0.025	0.001	0.007	0.011	0.001	0.000	0.000	0.000	0.000	0.001	0.000	0.000
15/2	0.885	0.082	0.000	0.002	0.026	0.001	0.002	0.003	0.000	0.000	0.000	0.000	0.000	0.000	0.000	0.000

Table S20. Squared magnitudes of projections of the local *ab initio* CF eigenstates calculated for the Dy3 ion of **2** onto pseudospin eigenstates with pseudospin J = 15/2 and projection M.

M	KD1		KD2		KD3		KD4		KD5		KD6		KD7		KD8	
−15/2	0.922	0.000	0.000	0.000	0.046	0.028	0.000	0.001	0.002	0.000	0.000	0.000	0.000	0.000	0.000	0.000
−13/2	0.000	0.000	0.607	0.336	0.007	0.005	0.013	0.006	0.021	0.000	0.001	0.001	0.002	0.000	0.000	0.000
−11/2	0.073	0.000	0.006	0.003	0.541	0.328	0.003	0.010	0.015	0.004	0.004	0.006	0.002	0.003	0.000	0.001
−9/2	0.003	0.000	0.026	0.014	0.010	0.005	0.198	0.083	0.618	0.009	0.011	0.005	0.003	0.012	0.002	0.001
−7/2	0.001	0.000	0.003	0.001	0.008	0.005	0.017	0.054	0.010	0.040	0.161	0.473	0.180	0.019	0.021	0.006
−5/2	0.000	0.000	0.002	0.001	0.002	0.001	0.105	0.045	0.040	0.003	0.141	0.024	0.099	0.384	0.027	0.127
−3/2	0.000	0.000	0.000	0.000	0.005	0.005	0.012	0.176	0.031	0.101	0.002	0.056	0.188	0.083	0.271	0.071
−1/2	0.000	0.000	0.000	0.000	0.005	0.001	0.247	0.028	0.105	0.002	0.079	0.035	0.014	0.012	0.107	0.365
1/2	0.000	0.000	0.000	0.000	0.001	0.005	0.028	0.247	0.002	0.105	0.035	0.079	0.012	0.014	0.365	0.107
3/2	0.000	0.000	0.000	0.000	0.005	0.005	0.176	0.012	0.101	0.031	0.056	0.002	0.083	0.188	0.071	0.271
5/2	0.000	0.000	0.001	0.002	0.001	0.002	0.045	0.105	0.003	0.040	0.024	0.141	0.384	0.099	0.127	0.027
7/2	0.000	0.001	0.001	0.003	0.005	0.008	0.054	0.017	0.040	0.010	0.473	0.161	0.019	0.180	0.006	0.021
9/2	0.000	0.003	0.014	0.026	0.005	0.010	0.083	0.198	0.009	0.618	0.005	0.011	0.012	0.003	0.001	0.002
11/2	0.000	0.073	0.003	0.006	0.328	0.541	0.010	0.003	0.004	0.015	0.006	0.004	0.003	0.002	0.001	0.000
13/2	0.000	0.000	0.336	0.607	0.005	0.007	0.006	0.013	0.000	0.021	0.001	0.001	0.000	0.002	0.000	0.000
15/2	0.000	0.922	0.000	0.000	0.028	0.046	0.001	0.000	0.000	0.002	0.000	0.000	0.000	0.000	0.000	0.000

Table S21. Squared magnitudes of projections of the local *ab initio* CF eigenstates calculated for the Dy1 ion of **3** onto pseudospin eigenstates with pseudospin J = 15/2 and projection M.

M	KD1		KD2		KD3		KD4		KD5		KD6		KD7		KD8	
−15/2	0.916	0.062	0.000	0.000	0.019	0.000	0.002	0.000	0.000	0.001	0.000	0.000	0.000	0.000	0.000	0.000
−13/2	0.000	0.000	0.011	0.905	0.002	0.003	0.004	0.050	0.017	0.003	0.004	0.000	0.000	0.001	0.000	0.000
−11/2	0.021	0.001	0.000	0.002	0.703	0.001	0.112	0.005	0.005	0.110	0.012	0.022	0.005	0.000	0.001	0.000
−9/2	0.000	0.000	0.001	0.072	0.007	0.007	0.013	0.362	0.312	0.027	0.134	0.023	0.003	0.033	0.000	0.006
−7/2	0.001	0.000	0.000	0.000	0.168	0.000	0.010	0.013	0.021	0.223	0.081	0.287	0.154	0.010	0.032	0.000
−5/2	0.000	0.000	0.000	0.006	0.001	0.016	0.019	0.214	0.000	0.004	0.187	0.046	0.027	0.353	0.000	0.127
−3/2	0.000	0.000	0.001	0.000	0.046	0.000	0.067	0.012	0.009	0.190	0.000	0.000	0.329	0.024	0.320	0.002
−1/2	0.000	0.000	0.000	0.002	0.000	0.025	0.012	0.106	0.074	0.004	0.167	0.036	0.008	0.054	0.000	0.510
1/2	0.000	0.000	0.002	0.000	0.025	0.000	0.106	0.012	0.004	0.074	0.036	0.167	0.054	0.008	0.510	0.000
3/2	0.000	0.000	0.000	0.001	0.000	0.046	0.012	0.067	0.190	0.009	0.000	0.000	0.024	0.329	0.002	0.320
5/2	0.000	0.000	0.006	0.000	0.016	0.001	0.214	0.019	0.004	0.000	0.046	0.187	0.353	0.027	0.127	0.000
7/2	0.000	0.001	0.000	0.000	0.000	0.168	0.013	0.010	0.223	0.021	0.287	0.081	0.010	0.154	0.000	0.032
9/2	0.000	0.000	0.072	0.001	0.007	0.007	0.362	0.013	0.027	0.312	0.023	0.134	0.033	0.003	0.006	0.000
11/2	0.001	0.021	0.002	0.000	0.001	0.703	0.005	0.112	0.110	0.005	0.022	0.012	0.000	0.005	0.000	0.001
13/2	0.000	0.000	0.905	0.011	0.003	0.002	0.050	0.004	0.003	0.017	0.000	0.004	0.001	0.000	0.000	0.000
15/2	0.062	0.916	0.000	0.000	0.000	0.019	0.000	0.002	0.001	0.000	0.000	0.000	0.000	0.000	0.000	0.000

Table S22. Squared magnitudes of projections of the local *ab initio* CF eigenstates calculated for the Dy2 ion of **3** onto pseudospin eigenstates with pseudospin J = 15/2 and projection M.

M	KD1		KD2		KD3		KD4		KD5		KD6		KD7		KD8	
−15/2	0.280	0.709	0.000	0.000	0.001	0.010	0.000	0.000	0.000	0.000	0.000	0.000	0.000	0.000	0.000	0.000
−13/2	0.000	0.000	0.182	0.806	0.000	0.003	0.000	0.008	0.000	0.000	0.000	0.000	0.000	0.000	0.000	0.000
−11/2	0.003	0.008	0.001	0.003	0.068	0.894	0.000	0.019	0.000	0.001	0.000	0.000	0.000	0.003	0.000	0.000
−9/2	0.000	0.000	0.001	0.006	0.002	0.017	0.017	0.929	0.002	0.011	0.003	0.000	0.010	0.001	0.000	0.000
−7/2	0.000	0.000	0.000	0.000	0.000	0.001	0.001	0.008	0.119	0.807	0.003	0.046	0.001	0.009	0.003	0.000
−5/2	0.000	0.000	0.000	0.001	0.000	0.000	0.000	0.006	0.037	0.008	0.778	0.102	0.028	0.001	0.009	0.029
−3/2	0.000	0.000	0.000	0.000	0.000	0.002	0.005	0.001	0.000	0.002	0.007	0.045	0.000	0.679	0.231	0.027
−1/2	0.000	0.000	0.000	0.000	0.001	0.000	0.000	0.005	0.009	0.004	0.010	0.003	0.267	0.000	0.089	0.611
1/2	0.000	0.000	0.000	0.000	0.000	0.001	0.005	0.000	0.004	0.009	0.003	0.010	0.000	0.267	0.611	0.089
3/2	0.000	0.000	0.000	0.000	0.002	0.000	0.001	0.005	0.002	0.000	0.045	0.007	0.679	0.000	0.027	0.231
5/2	0.000	0.000	0.001	0.000	0.000	0.000	0.006	0.000	0.008	0.037	0.102	0.778	0.001	0.028	0.029	0.009
7/2	0.000	0.000	0.000	0.000	0.001	0.000	0.008	0.001	0.807	0.119	0.046	0.003	0.009	0.001	0.000	0.003
9/2	0.000	0.000	0.006	0.001	0.017	0.002	0.929	0.017	0.011	0.002	0.000	0.003	0.001	0.010	0.000	0.000
11/2	0.008	0.003	0.003	0.001	0.894	0.068	0.019	0.000	0.001	0.000	0.000	0.000	0.003	0.000	0.000	0.000
13/2	0.000	0.000	0.806	0.182	0.003	0.000	0.008	0.000	0.000	0.000	0.000	0.000	0.000	0.000	0.000	0.000
15/2	0.709	0.280	0.000	0.000	0.010	0.001	0.000	0.000	0.000	0.000	0.000	0.000	0.000	0.000	0.000	0.000

Table S23. Squared magnitudes of projections of the local *ab initio* CF eigenstates calculated for the Dy3 ion of **3** onto pseudospin eigenstates with pseudospin J = 15/2 and projection M.

M	KD1		KD2		KD3		KD4		KD5		KD6		KD7		KD8	
−15/2	0.871	0.108	0.000	0.000	0.000	0.018	0.000	0.001	0.001	0.000	0.000	0.000	0.000	0.000	0.000	0.000
−13/2	0.000	0.000	0.857	0.055	0.003	0.001	0.047	0.013	0.006	0.012	0.004	0.000	0.000	0.000	0.000	0.000
−11/2	0.018	0.002	0.000	0.000	0.001	0.706	0.021	0.086	0.091	0.035	0.002	0.032	0.004	0.001	0.001	0.000
−9/2	0.000	0.000	0.074	0.005	0.007	0.001	0.323	0.081	0.093	0.215	0.147	0.012	0.008	0.028	0.001	0.005
−7/2	0.000	0.000	0.000	0.000	0.000	0.180	0.006	0.008	0.174	0.087	0.028	0.324	0.111	0.050	0.030	0.002
−5/2	0.000	0.000	0.006	0.000	0.015	0.001	0.189	0.048	0.000	0.001	0.221	0.022	0.098	0.275	0.005	0.119
−3/2	0.000	0.000	0.000	0.001	0.000	0.045	0.019	0.049	0.146	0.058	0.001	0.000	0.257	0.101	0.303	0.017
−1/2	0.000	0.000	0.002	0.000	0.023	0.000	0.084	0.025	0.017	0.062	0.185	0.021	0.023	0.042	0.024	0.493
1/2	0.000	0.000	0.000	0.002	0.000	0.023	0.025	0.084	0.062	0.017	0.021	0.185	0.042	0.023	0.493	0.024
3/2	0.000	0.000	0.001	0.000	0.045	0.000	0.049	0.019	0.058	0.146	0.000	0.001	0.101	0.257	0.017	0.303
5/2	0.000	0.000	0.000	0.006	0.001	0.015	0.048	0.189	0.001	0.000	0.022	0.221	0.275	0.098	0.119	0.005
7/2	0.000	0.000	0.000	0.000	0.180	0.000	0.008	0.006	0.087	0.174	0.324	0.028	0.050	0.111	0.002	0.030
9/2	0.000	0.000	0.005	0.074	0.001	0.007	0.081	0.323	0.215	0.093	0.012	0.147	0.028	0.008	0.005	0.001
11/2	0.002	0.018	0.000	0.000	0.706	0.001	0.086	0.021	0.035	0.091	0.032	0.002	0.001	0.004	0.000	0.001
13/2	0.000	0.000	0.055	0.857	0.001	0.003	0.013	0.047	0.012	0.006	0.000	0.004	0.000	0.000	0.000	0.000
15/2	0.108	0.871	0.000	0.000	0.018	0.000	0.001	0.000	0.000	0.001	0.000	0.000	0.000	0.000	0.000	0.000

Table S24. Magnitudes of the local transition magnetic moment matrix elements (in units Bohr magneton) calculated for the Dy1 ion of **2**.

Inital KD	Final KD	Climbing transition	Crossing transition
1	1	3.266660	0.000959
1	2	1.786833	0.001819
1	3	0.345548	0.001107
1	4	0.348636	0.017785
1	5	0.131364	0.062762
1	6	0.097077	0.026056
1	7	0.038087	0.016545
1	8	0.010207	0.005308
2	2	3.052568	0.007087
2	3	2.318168	0.011579
2	4	0.296779	0.052973
2	5	0.260257	0.046471
2	6	0.304932	0.039731
2	7	0.100225	0.120524
2	8	0.042240	0.042384
3	3	2.704168	0.028318
3	4	2.649591	0.126229
3	5	0.686681	0.486069
3	6	0.374302	0.132817
3	7	0.072350	0.109489
3	8	0.066879	0.073176
4	4	2.434880	0.806587
4	5	2.160610	1.251936
4	6	1.980737	0.541019
4	7	0.278396	0.642315
4	8	0.119301	0.184130
5	5	3.135840	1.085268
5	6	2.610921	1.075214
5	7	0.756150	0.486212
5	8	0.191298	0.153437
6	6	1.642566	2.421725
6	7	2.407913	1.291370
6	8	0.420615	0.365154
7	7	0.968136	3.015850
7	8	1.412202	1.110728
8	8	3.140742	1.235175

Table S25. Magnitudes of the local transition magnetic moment matrix elements (in units Bohr magneton) calculated for the Dy2 ion of **2**.

Inital KD	Final KD	Climbing transition	Crossing transition
1	1	3.285109	0.000139
1	2	1.724721	0.000455
1	3	0.700119	0.000903
1	4	0.228159	0.001942
1	5	0.148777	0.013739
1	6	0.021138	0.035442
1	7	0.046347	0.008299
1	8	0.021715	0.003816
2	2	3.507741	0.001359
2	3	2.210352	0.003827
2	4	0.749884	0.009026
2	5	0.245151	0.030174
2	6	0.184623	0.054600
2	7	0.131969	0.023324
2	8	0.042100	0.018076
3	3	3.505097	0.006387
3	4	2.877871	0.026444
3	5	0.276906	0.091174
3	6	0.191222	0.145453
3	7	0.200480	0.150053
3	8	0.093144	0.088093
4	4	2.110242	0.067661
4	5	3.210532	0.194899
4	6	0.523027	0.318249
4	7	0.481564	0.527963
4	8	0.151782	0.340769
5	5	1.795214	0.664431
5	6	2.628207	1.132095
5	7	1.764951	1.207141
5	8	0.469285	0.724290
6	6	0.908984	2.963666
6	7	1.787604	1.640774
6	8	0.616304	0.540548
7	7	3.090107	1.185870
7	8	1.865730	0.445639
8	8	3.282979	0.936497

Table S26. Magnitudes of the local transition magnetic moment matrix elements (in units Bohr magneton) calculated for the Dy3 ion of **2**.

Inital KD	Final KD	Climbing transition	Crossing transition
1	1	3.241920	0.000893
1	2	1.814064	0.001712
1	3	0.456768	0.006813
1	4	0.270692	0.067727
1	5	0.445087	0.025848
1	6	0.088651	0.054890
1	7	0.035517	0.049931
1	8	0.012577	0.015055
2	2	3.179362	0.004736
2	3	2.313304	0.012527
2	4	0.333145	0.082981
2	5	0.191612	0.086531
2	6	0.364080	0.046058
2	7	0.178554	0.138382
2	8	0.027593	0.065588
3	3	2.693554	0.111278
3	4	1.980423	0.478235
3	5	2.026412	0.247730
3	6	0.302094	0.227618
3	7	0.204105	0.107012
3	8	0.058599	0.100582
4	4	2.527558	2.205824
4	5	2.033110	1.654593
4	6	1.388338	0.613736
4	7	0.535573	0.491701
4	8	0.174369	0.195216
5	5	2.298517	1.318101
5	6	2.787102	0.699715
5	7	0.894243	0.793340
5	8	0.130719	0.293315
6	6	1.034666	2.798347
6	7	2.008216	1.573236
6	8	0.331136	0.237259
7	7	2.393122	1.781507
7	8	1.087883	1.535400
8	8	1.532699	3.368560

Table S27. Magnitudes of the local transition magnetic moment matrix elements (in units Bohr magneton) calculated for the Dy1 ion of **3**.

Inital KD	Final KD	Climbing transition	Crossing transition
1	1	3.293912	0.001423
1	2	1.788662	0.005327
1	3	0.229221	0.043663
1	4	0.098477	0.051844
1	5	0.033264	0.030731
1	6	0.025082	0.025274
1	7	0.015431	0.012860
1	8	0.004140	0.004208
2	2	2.939210	0.067861
2	3	2.380760	0.137444
2	4	0.352133	0.495210
2	5	0.259765	0.123813
2	6	0.059036	0.034904
2	7	0.044327	0.043291
2	8	0.016592	0.016257
3	3	2.239086	0.995955
3	4	2.601380	0.907333
3	5	0.689510	0.776441
3	6	0.101981	0.173605
3	7	0.051112	0.032017
3	8	0.026036	0.029622
4	4	0.953523	2.193268
4	5	2.239680	2.118662
4	6	0.338017	0.319245
4	7	0.067986	0.095205
4	8	0.025670	0.027307
5	5	1.783329	0.999828
5	6	2.716547	0.833343
5	7	0.463612	0.127391
5	8	0.036093	0.138153
6	6	2.476806	0.805591
6	7	2.399175	0.331624
6	8	0.047540	0.267216
7	7	2.857466	0.719793
7	8	0.216052	1.721432
8	8	3.347148	0.023864

Table S28. Magnitudes of the local transition magnetic moment matrix elements (in units Bohr magneton) calculated for the Dy2 ion of **3**.

Inital KD	Final KD	Climbing transition	Crossing transition
1	1	3.306052	0.000264
1	2	1.771009	0.000690
1	3	0.226559	0.001273
1	4	0.171370	0.002227
1	5	0.047177	0.015135
1	6	0.028117	0.005105
1	7	0.022247	0.021918
1	8	0.008119	0.005726
2	2	3.020950	0.008691
2	3	2.344935	0.011826
2	4	0.251190	0.016064
2	5	0.297673	0.030763
2	6	0.076433	0.054599
2	7	0.066445	0.041069
2	8	0.041990	0.034965
3	3	2.753247	0.072531
3	4	2.818603	0.074602
3	5	0.201865	0.118335
3	6	0.144213	0.070800
3	7	0.132653	0.117294
3	8	0.077086	0.067614
4	4	2.091379	0.236955
4	5	3.052398	0.214138
4	6	0.251699	0.492844
4	7	0.213808	0.236801
4	8	0.123918	0.114958
5	5	1.613249	1.372905
5	6	2.963819	0.613831
5	7	0.289768	0.542225
5	8	0.470276	0.206439
6	6	0.974852	1.884965
6	7	2.799384	0.449991
6	8	0.939763	1.358272
7	7	0.484118	3.499799
7	8	1.609279	1.127629
8	8	3.620927	2.497399

Table S29. Magnitudes of the local transition magnetic moment matrix elements (in units Bohr magneton) calculated for the Dy3 ion of **3**.

Inital KD	Final KD	Climbing transition	Crossing transition
1	1	3.295232	0.001223
1	2	1.784586	0.004545
1	3	0.181092	0.046686
1	4	0.109189	0.035720
1	5	0.026653	0.022150
1	6	0.010241	0.024981
1	7	0.006457	0.017078
1	8	0.002091	0.005146
2	2	2.850948	0.069302
2	3	2.382280	0.132526
2	4	0.295654	0.473545
2	5	0.233246	0.093887
2	6	0.012758	0.032994
2	7	0.020080	0.054320
2	8	0.007806	0.020805
3	3	2.120967	0.973201
3	4	2.619910	0.860169
3	5	0.362965	0.799300
3	6	0.107331	0.096214
3	7	0.022890	0.056548
3	8	0.014602	0.038650
4	4	1.046680	2.080416
4	5	2.085298	1.392481
4	6	0.269233	0.394168
4	7	0.035900	0.022817
4	8	0.019813	0.036245
5	5	0.360218	2.011958
5	6	1.924102	1.933985
5	7	0.234658	0.259346
5	8	0.061942	0.067924
6	6	2.027740	1.169943
6	7	2.312610	0.613579
6	8	0.218712	0.060886
7	7	2.869677	0.155568
7	8	1.725652	0.045114
8	8	3.289451	0.046377

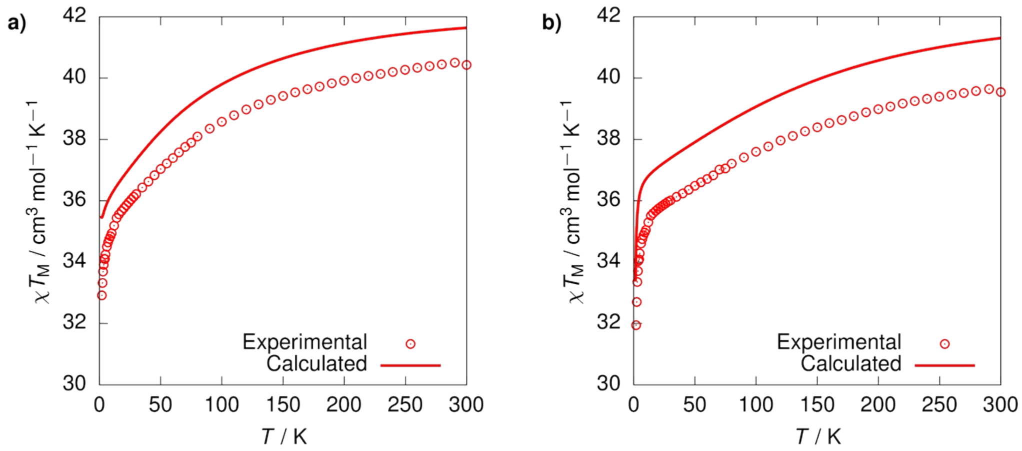

Fig. S20. Calculated and measured magnetic susceptibilites of **2** (left) and **3** (right). The deviation between the calculated and experimental values are approximately 3-4%.

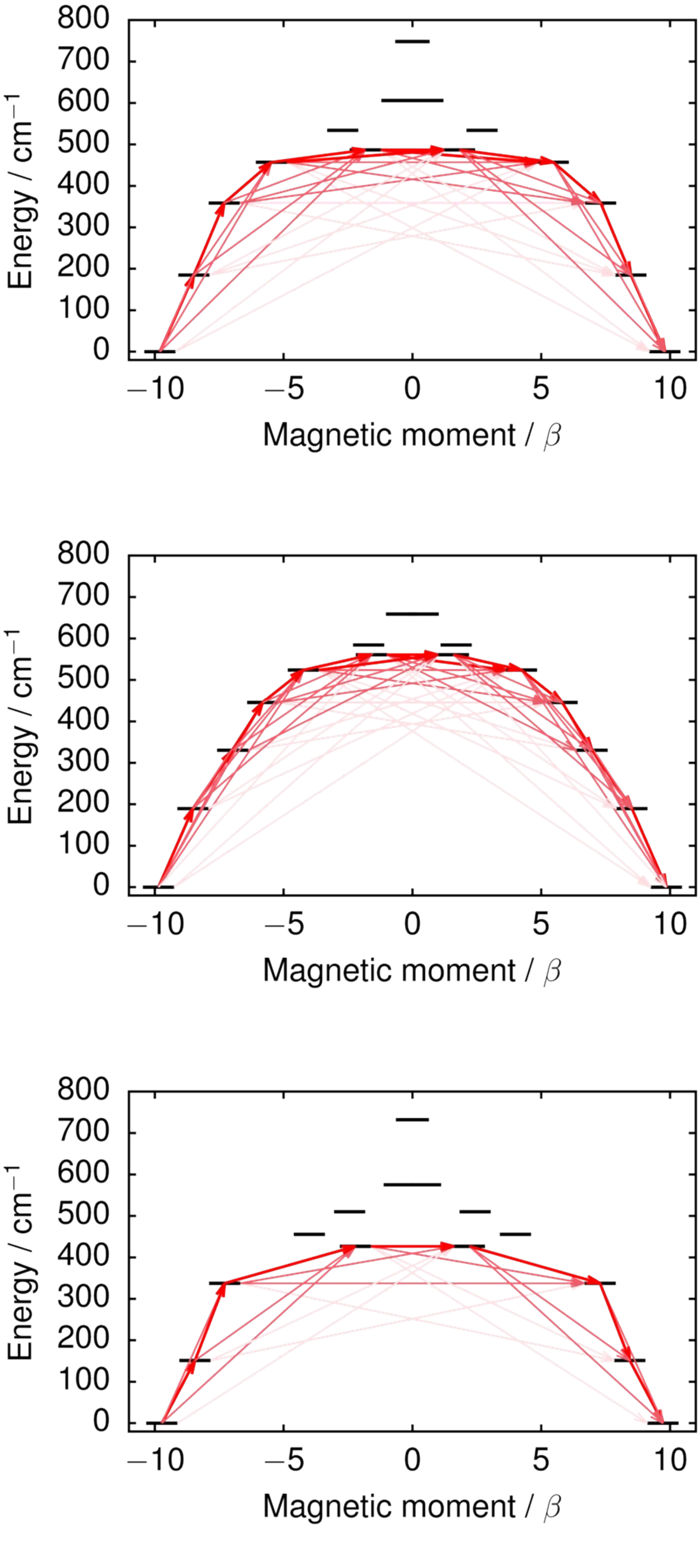

Fig. S21. Calculated local effective *ab initio* barriers for the relaxation of magnetization at the Dy1 (top), Dy2 (middle) and Dy3 (bottom) ions of **2**. Stronger arrows indicate larger absolute value of the transition magnetic moment matrix elements between the respective states. Transitions involving higher-energy states not involved in the relaxation mechanism are omitted for clarity.

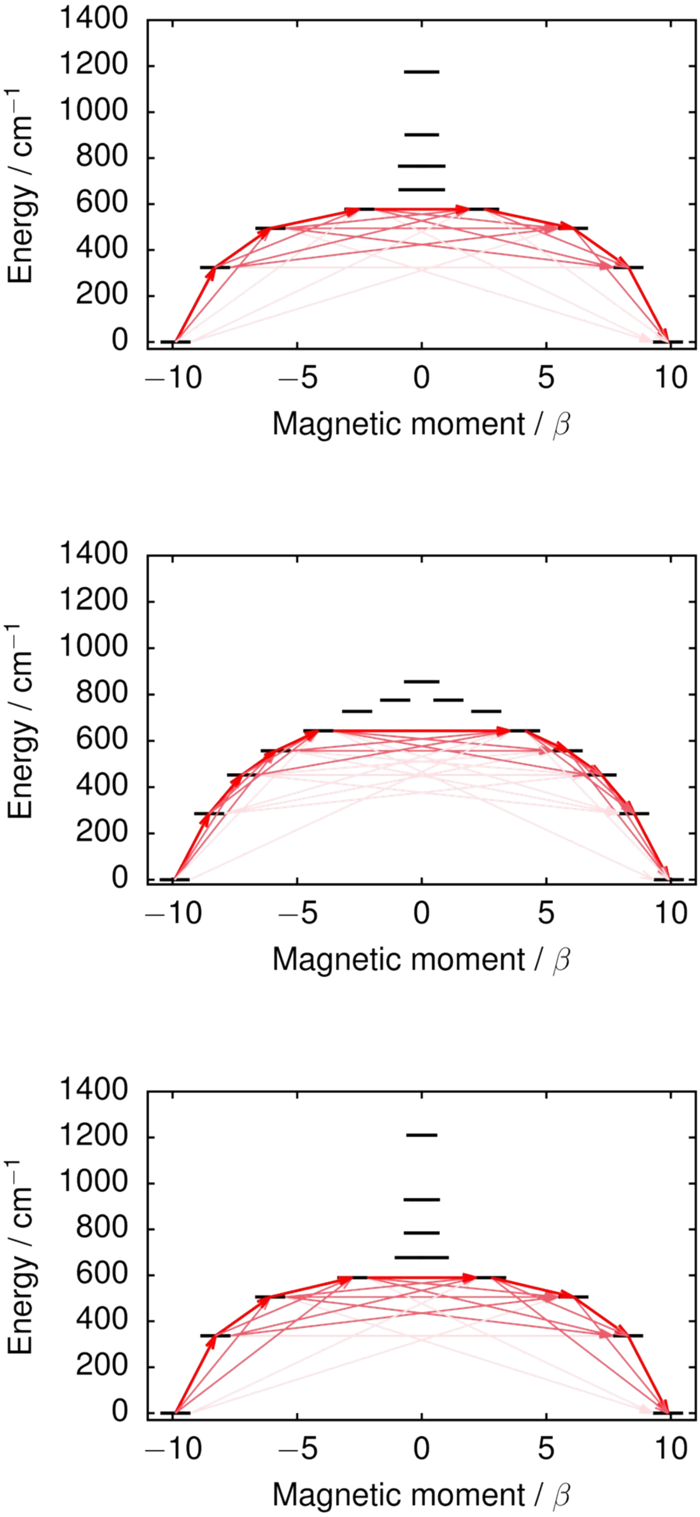

Fig. S22. Calculated local effective *ab initio* barriers for the relaxation of magnetization at the Dy1 (top), Dy2 (middle) and Dy3 (bottom) ions of **3**. Stronger arrows indicate larger absolute value of the transition magnetic moment matrix elements between the respective states. Transitions involving higher-energy states not involved in the relaxation mechanism are omitted for clarity.

References:

1. M. He; F. S. Guo; J. Tang; A. Mansikkamaki; R. A. Layfield, *Chem Sci* **2020,** *11*, 5745-5752.
2. O. V. Dolomanov, L. J. Bourhis, R. J. Gildea, J. A. K. Howard, H. Puschmann, *J. Appl. Cryst.* **2009**, 42, 339- 341.
3. G. M. Sheldrick, *Acta Cryst.* **2015**, A71, 3-8.
4. G. M. Sheldrick, *Acta Cryst.* **2015**, C71, 3-8.
5. D. Gatteschi, R. Sessoli, J. Villain, *Molecular Nanomagnets* (Oxford Univ. Press, 2006).
6. a) *ADF2019*. SCM, Theoretical Chemistry, Vrije Universiteit Amsterdam, The Neatherlands. http://www.scm.com. 2019; b) G. te Velde, F. M. Bickelhaupt, E. J. Baerendes, C. Fonseca Guerra, S. J. A. Gisbergen, J. G. Snijders, T. Ziegler. *J. Comp. Chem.* **2001**, *22*, 931–967; c) C. Fonseca Guerra, J. G. Snijders, G. te Velde, E. J. Baerends. *Theor. Chem. Acc.* **1998**, *99*, 391–403.
7. a) J. P. Perdew, K. Burke, M. Ernzerhof. *Phys. Rev. Lett.*, **1996**, *77*, 3865–3868; b) J. P. Perdew, K. Burke, M. Ernzerhof. *Phys. Rev. Lett.*, **1996**, *78*, 1396.
8. S. Grimme, J. Antony, S. Ehrlich, H. Krieg. *J. Chem. Phys.* **2010**, *132*, 154104.
9. S. Grimme, S. Ehrlich, L. Goerigk. *J. Comp. Chem.* **2011**, *32*, 1456–1465.
10. a) E. van Lenthe, E. J. Baerends, J. G. Snijders. *J. Chem. Phys.* **1993**, *99*, 4597–4610; b) E. van Lenthe, E. J. Baerends, J. G. Snijders. *J. Chem. Phys.* **1994**, *101*, 9783–9792; c) E. van Lenthe, R. van Leeuwen, E. J. Baerends, J. G. Snijders. *Int. J. Quantum. Chem.* **1996**, *57*, 281–293.
11. E. van Lenthe, E. J. Baerends. *J. Comp. Chem.* **2003**, *24*, 1142–1156.
12. a) I. F. Galván, M. Vacher, A. Alavi, C. Angeli, F. Aquilante, J. Autschbach, J. J. Bao, S. I. Bokarev, N. A. Bogdanov, R. K. Carlson, L. F. Chibotaru, J. Creutzberg, N. Dattani, M. G. Delcey, S. S. Dong, A. Dreuw, L. Freitag, L. M. Frutos, L. Gagliardi, F. Gendron, A. Giussani, L. González, G. Grell, M. Guo, C. E. Hoyer, M. Johansson, S. Keller, S. Knecht, G. Kovačević, E. Källman, G. L. Manni, M. Lundberg, Y. Ma, S. Mai, J. P. Malhado, P. Å. Malmqvist, P. Marquetand, S. A. Mewes, J. Norell, M. Olivucci, M. Oppel, Q. M. Phung, K. Pierloot, F. Plasser, M. Reiher, A. M. Sand, I. Schapiro, P. Sharma, C. J. Stein, L. K. Sørensen, D. G. Truhlar, M. Ugandi, L. Ungur, A. Valentini, S. Vancoillie, V. Veryazov, O. Weser, T. A. Wesołowski, P.-O. Widmark, S. Wouters, A. Zech, J. P. Zobel, R. Lindh. *J. Chem. Theory Comput.*, **2019**, *15*, 5925–5964; b) F. Aquilante, J. Autschbach, A. Baiardi, S. Battaglia, V. A. Borin, Veniamin, L. F. Chibotaru, I. Conti, L. De Vico, M. Delcey, I. Fdez. Galván, N. Ferré, L. Freitag, M. Garavelli, X. Gong, S. Knecht, E. D. Larsson, R. Lindh, M. Lundberg, P. Å. Malmqvist, A. Nenov, J. Norell, M. Odelius, M. Olivucci, T. B. Pedersen, L. Pedraza-González, Q. M. Phung, K. Pierloot, M. Reiher, I. Schapiro, J. Segarra-Mart\'i, F. Segatta, L. Seijo, S. Sen, D.-C. Sergentu, C. J. Stein, L. Ungur, M. Vacher, A. Valentini, V. Veryazov. *J. Chem. Phys.* **2020**, *152*, 214117.
13. a) B. O. Roos in *Advances in Chemical Physics*, *Ab Initio Methods in Quantum Chemistry II*, Vol. 69 (Ed.: K. P. Lawley), Wiley, New York, **1987**, pp. 399–455; b) P. Siegbahn, A. Heiberg, B. Roos, B. Levy. *Phys. Scripta*, **1980**, *21*, 323–327; c) B. O. Roos, P. R. Taylor, P. E. M. Siegbahn. *Chem. Phys.*, **1980**, *48*, 157–173; d) P. E. M. Siegbahn, J. Almlöf, A. Heiberg, B. Roos. *J. Chem. Phys.*, **1981**, *74*, 2384–2396; e) B. O. Roos, R. Lindh, P. Å. Malmqvist, V. Veryazov, P.-O. Widmark. *Multiconfigurational Quantum Chemistry.* Wiley, Hoboken, NJ, **2016**.
14. P. Å. Malmqvist, B. O. Roos, B. Schimmelpfenning. *Chem. Phys. Lett.*, **2002**, *357*, 230–240.
15. a) B. A. Heß, C. M. Marian, U. Wahlgren, O. Gropen. *Chem. Phys. Lett.*, **1996**, *251*, 365–371; b) O. Christiansen, J. Gauss, B. Schimmelpfennig. *Phys. Chem. Chem. Phys.*, **2000**, *2*, 965–971.
16. a) L. F. Chibotaru, L. Ungur. *J. Chem. Phys.*, **2012**, *137*, 064112; b) L. Ungur, L. F. Chibotaru. *Chem. Eur. J.* **2017**, *23*, 3708–3718; c) L. Ungur, M. Thewissen, J.-P. Costes, W. Wernsdorfer, L. F. Chibotaru. *Inorg. Chem.*, **2013**, *52*, 6328–6337.
17. L. Ungur, L. F. Chibotaru. Computational *Modelling of Magnetic Properties of Lanthanide Compounds* in *Lanthanide and Actinides in Molecular Magnetism.* Eds. R. A. Layfield, M. Murugesu. Wiley,-VHC, Weinheim, Germany, **2015**.
18. a) P.-O. Widmark, P.-Å. Malmqvist, B. O. Roos. *Theor. Chim. Acta*, **1990**, *77*, 1432–2234; b) B. O. Roos, R. Lindh, P.-Å. Malmqvist, V. Veryazov, P.-O. Widmark. *J. Phys. Chem. A*, **2004**, *108*, 2851–2858; c) B. O. Roos, R. Lindh, P.-Å. Malmqvist, V. Veryazov, P.-O. Widmark. *J. Phys. Chem. A*, **2005**, *109*, 6575–6579; d) B. O. Roos, R. Lindh, P.-Å. Malmqvist, V. Veryazov, P.-O. Widmark, A. C. Borin. *J. Phys. Chem. A.* **2008**, *112*, 11431–11435.
19. a) W. Kutzelnigg, W. Liu. *J. Chem. Phys.*, **2005**, *123*, 241102; b) M. Filatov. *J. Chem. Phys.* **2006**, *125*, 107101; c) . Daoling, M. Reiher. *Theor. Chem. Acc.,* **2012**, *131*, 1.
20. a) L. F. Chibotaru, L. Ungur, A. Soncini. *Angew. Chem.* **2008**, *120*, 4194–4197; b) L. Ungur, W. van den Heuvel, L. F. Chibotaru. *New. J. Chem.* **2009**, *33*, 1223–1230.

21. a) N. Iwahara, L. F. Chibotaru. *Phys. Rev. B.* **2015**, *91*, 174438; b) N. Iwahara, L. Ungur, L. F. Chibotaru. *Phys. Rev. B.*, **2018**, *98*, 054436.
